# The impact of moderator by confounder interactions in the assessment of treatment effect modification: a simulation study

**DOI:** 10.1186/s12874-022-01519-7

**Published:** 2022-04-03

**Authors:** Antonia Mary Marsden, William G. Dixon, Graham Dunn, Richard Emsley

**Affiliations:** 1 Centre for Biostatistics, School of Health Sciences, The University of Manchester, Manchester Academic Health Science Centre, Jean McFarlane Building, Oxford Road, Manchester, M13 9PL UK; 2 Centre for Epidemiology Versus Arthritis, Manchester Academic Health Science Centre, University of Manchester, Manchester, UK; 3 Institute of Psychiatry, King’s College London, Psychology & Neuroscience, London, UK

**Keywords:** Confounding, Interaction, Propensity scores, Treatment effect modification

## Abstract

**Background:**

When performed in an observational setting, treatment effect modification analyses should account for all confounding, where possible. Often, such studies only consider confounding between the exposure and outcome. However, there is scope for misspecification of the confounding adjustment when estimating moderation as the effects of the confounders may themselves be influenced by the moderator. The aim of this study was to investigate bias in estimates of treatment effect modification resulting from failure to account for an interaction between a binary moderator and a confounder on either treatment receipt or the outcome, and to assess the performance of different approaches to account for such interactions.

**Methods:**

The theory behind the reason for bias and factors that impact the magnitude of bias is explained. Monte Carlo simulations were used to assess the performance of different propensity scores adjustment methods and regression adjustment where the adjustment 1) did not account for any moderator-confounder interactions, 2) included moderator-confounder interactions, and 3) was estimated separately in each moderator subgroup. A real-world observational dataset was used to demonstrate this issue.

**Results:**

Regression adjustment and propensity score covariate adjustment were sensitive to the presence of moderator-confounder interactions on outcome, whilst propensity score weighting and matching were more sensitive to the presence of moderator-confounder interactions on treatment receipt. Including the relevant moderator-confounder interactions in the propensity score (for methods using this) or the outcome model (for regression adjustment) rectified this for all methods except propensity score covariate adjustment. For the latter, subgroup-specific propensity scores were required. Analysis of the real-world dataset showed that accounting for a moderator-confounder interaction can change the estimate of effect modification.

**Conclusions:**

When estimating treatment effect modification whilst adjusting for confounders, moderator-confounder interactions on outcome or treatment receipt should be accounted for.

**Supplementary Information:**

The online version contains supplementary material available at 10.1186/s12874-022-01519-7.

## Introduction

Treatment effect modification (TEM) occurs when the effect of treatment on an outcome is influenced by a third variable, termed a moderator. Such an analysis can identify patients who are more likely to benefit or be harmed from treatment. In some cases, a moderator may have a strong scientific-rationale and their investigation is pre-specified. For example, in their randomised controlled trial protocol, Kyle et al. hypothesised that age may moderate the effect of cognitive behavioural therapy for insomnia on cognitive functioning outcome [1]. In other cases, researchers may investigate several potential moderators in an exploratory manner at the analysis stage. TEM is typically evaluated by including a product term (statistical interaction) between treatment and the moderator in a regression model applied to the full cohort of patients in the study.

Randomised clinical trials provide the best evidence regarding the causal effects of treatments, and thus also the best evidence for the existence of TEM, although if the causal effect of a moderator is of interest, treatment randomisation does not ensure unbiased estimation of this [2]. Observational studies however are more feasible for many research questions, for example when investigating rare treatment side-effects or assessing real-world effectiveness. Observational studies require appropriate adjustment of confounders in order for valid inference on the causal effect of treatments to be made [3]. Confounders are often accounted for via regression adjustment in a multivariable regression model or, increasingly, by the use of propensity scores [4].

Observational studies attempt to adjust for confounding of the treatment–outcome relationship but often do not consider any additional features required to unbiasedly evaluate TEM [5]. Here, we focus on the situation where the moderator not only influences the relationship between the treatment and outcome, but also the relationship between a confounder and either treatment receipt or the outcome.

**Fig. 1:**
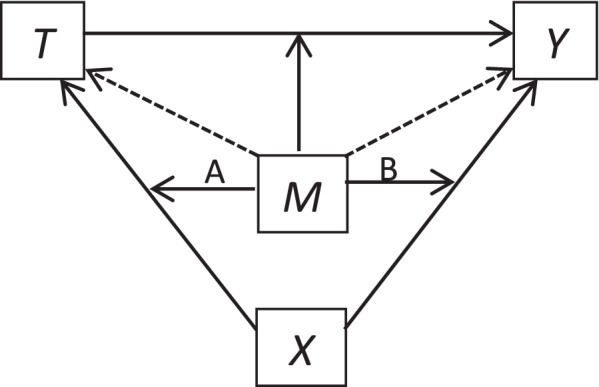
Graphical representation of a moderator which influences the confounder-exposure and confounder-outcome relationships. The moderator, ${\varvec{M}}$, by definition influences the relationship between the exposure, ${\varvec{T}}$, and outcome, ${\varvec{Y}}$, but also may influence the relationship between a confounder, ${\varvec{X}}$, and the exposure and/or the relationship between a confounder and the outcome. The moderator may also be a confounder between the exposure and outcome (shown via a dotted line), but not necessarily

Figure 1 illustrates the concept where path A represents the moderator influencing the effect of a confounder on treatment receipt and path B represents the moderator influencing the effect of a confounder on the outcome. If the moderator influences the effect of the confounder on treatment receipt, this implies that the way in which the confounder influences the decision to prescribe treatment varies across the moderator, e.g. obesity (X) may discourage clinicians from prescribing a specific treatment (T) in women more than in men (M). If the moderator influences the effect of the confounder on outcome, this implies that the relationship between the confounder and the outcome varies across the moderator, e.g. obesity (X) may increase the risk of heart disease (Y) by a larger amount in men than in women (M). In many cases, the moderator will itself be a confounder – although not necessarily. For example, a treatment may be more effective in reducing cardiovascular disease in older people than in younger people, (i.e. age moderates the effect of treatment), but age would only also be a confounder if it were associated with both receipt of treatment and the outcome.

If the differential effect of the confounder across the moderator is not accounted for, this may introduce bias into the estimate of treatment effect modification. Suppose M is binary and the effect of the confounder is greater in one subgroup of M than the other. Accounting for the overall effect of the confounder will lead to an underestimation of the effect of the confounder in one subgroup and an overestimation of the effect of the confounder in the other subgroup, which will lead to biased estimates of the subgroup treatment effects, and hence treatment effect modification. The magnitude of the bias will depend on the prevalence of the moderator, the relative sizes of the moderator and confounder effects, and the number of confounders which are influenced by the moderator. A more detailed explanation is given in the Supplementary material.

Failure to account for an interaction that exists between a moderator and confounder is essentially a misspecification problem. Both propensity score methods and regression adjustment are sensitive to model misspecification. Some research has indicated regression adjustment (without PS methods) is more sensitive to model specification than PS methods [6]. Furthermore, there is variation amongst PS methods: Greifer and Stuart say that matching methods are less sensitive to misspecification than weighting methods, as the former does not directly rely on the exact propensity score [7]. In practice, whether or not PS methods or regression adjustment will perform better under the corresponding model misspecification will likely vary by study, depending on how complex or well understood the treatment model or outcome model is and how much information is available to model each.

Consideration of differences in treatment assignment across subgroups of a moderator when applying propensity score (PS) methods to estimate subgroup-specific effects has been discussed previously [8–11, 12]. Radice et al. [9] and Krief et al. [8] showed that using subgroup-specific propensity scores for PS matching and inverse probability of treatment weighting (IPTW) resulted in better covariate balance and lower bias than when differences in treatment assignment were ignored. Wang et al. [11] and Green and Stuart [10] focussed on different PS matching techniques and similarly found that balance metrics were improved when differences in treatment assignment were accounted for, either by estimating subgroup-specific PS models or including moderator-confounder interactions in a single PS model.

In this paper, we aim to add to this literature by additionally considering 1) PS covariate adjustment and regression adjustment as confounding adjustment methods, 2) situations where the moderator influences the relationship between the confounder and either/both the treatment (or exposure) and outcome, and 3) bias introduced into estimates of both TEM and subgroup-specific treatment effects. Patterns of bias under these different scenarios are explored in a simulation study. We discuss factors that influence the magnitude of bias and compare reduction in bias and precision of different approaches to accounting for moderator-confounder interactions.

To investigate the impact of the issues discussed in practice, we compared the estimates of treatment effect modification in a real observational dataset when moderator-confounder interactions were and were not included in the confounding adjustment.  The dataset comprised information from the 2018-19 National Survey for Wales and the interest was in whether the effect of tinnitus on mental well-being was moderated by certain variables. 

## Methods

### Simulation study

We performed a simulation study to confirm and demonstrate that 1) estimates of subgroup-specific treatment effects and TEM can be biased if the moderator also influences the effect of the confounders and this is unaccounted for, 2) the presence of bias depends on the confounding adjustment method and whether or not the moderator influences the effect of the confounder on treatment receipt or the effect of the confounder on the outcome, and 3) the impact of this bias depends on the prevalence of the moderator and the relative effect sizes of the treatment effect moderation. We compared the accuracy and precision of estimates between methods of accounting for the moderator-confounder interactions.

#### Data generation

The simulated data comprised the following information on each patient: $T$ – a binary indicator of treatment assignment (yes/no), $Y$ - a continuous outcome measure, $M$ – a binary treatment effect moderator, ${X}_{1}, {X}_{2}, {X}_{3}$—three continuous confounding variables, and ${X}_{4}, {X}_{5}, {X}_{6}$ – three binary confounding variables. Throughout this paper, we assume no treatment-induced confounding [13]. Datasets of size 1000 were generated to reflect a moderately large but realistic sample size and to allow patterns in the magnitude of the standard errors to be more easily assessed graphically.

The three continuous confounders ${X}_{1}, {X}_{2}, {X}_{3}$ were defined to follow a $N(\mathrm{0,1})$ distribution. Binary confounders ${X}_{4}, {X}_{5}, {X}_{6}$ were defined to have prevalence 0.1, 0.25 and 0.5 respectively. $M$ was simulated first with prevalence 0.5 and then 0.1.

An individual’s true probability of treatment was defined to depend on the main effects of the variables ${X}_{1}, \dots ,{X}_{6}, M$ and a product term between $M$ and ${X}_{1}$ representing modification of the effect of ${X}_{1}$ on treatment receipt by $M$:

$$\mathrm{log}\left(\frac{p}{1-p}\right)={\alpha }_{0}+{\alpha }_{M}M+{\alpha }_{M{X}_{1}}M{X}_{1}+\sum_{k=1}^{6}{\alpha }_{{X}_{j}}{X}_{j}+{e}_{1}$$

$p$ is the probability of treatment allocation (i.e. $P(T=1|{\varvec{X}}, M)$), and ${e}_{1}\sim N(0, 0.2)$. The chosen values of the $\alpha$ coefficients are given in Table 1. The binary treatment variable, $T$, was generated via a Bernoulli distribution: $T\sim \mathrm{Bernoulli}(p)$.

**Table 1:** Model coefficient values in the data generation models in the simulation study. The values quantify the effects of the model covariates on both the probability of treatment allocation and the outcome. Three different values for the ${\varvec{T}}\times {\varvec{M}}$ term in the outcome model, and the ${\varvec{M}}\times {{\varvec{X}}}_{1}$ term in the probability of treatment model and the outcome model were considered

	**Propensity Score Model**		**Outcome model**	
	**Notation**	**Value**	**Notation**	**Value**
**Intercept**	${\alpha }_{0}$	0.1	${\beta }_{0}$	0.25
${\varvec{T}}$			${\beta }_{T}$	1.5
${\varvec{M}}$	${\alpha }_{M}$	0.3	${\beta }_{M}$	0.5
${\varvec{T}}\times {\varvec{M}}$			${\beta }_{TM}$	(0.3, 0.6)
${{\varvec{X}}}_{1}$	${\alpha }_{{X}_{1}}$	0.3	${\beta }_{{X}_{1}}$	0.5
${\varvec{M}}\times {{\varvec{X}}}_{1}$	${\alpha }_{M{X}_{1}}$	(0, 0.1, 0.2)	${\beta }_{M{X}_{1}}$	(0, 0.2, 0.4)
${{\varvec{X}}}_{2}$	${\alpha }_{{X}_{2}}$	0.2	${\beta }_{{X}_{2}}$	0.5
${{\varvec{X}}}_{3}$	${\alpha }_{{X}_{3}}$	-0.1	${\beta }_{{X}_{3}}$	-0.3
${{\varvec{X}}}_{4}$	${\alpha }_{{X}_{4}}$	0.4	${\beta }_{{X}_{4}}$	1
${{\varvec{X}}}_{5}$	${\alpha }_{{X}_{5}}$	-0.2	${\beta }_{{X}_{5}}$	0.6
${{\varvec{X}}}_{6}$	${\alpha }_{{X}_{6}}$	0.3	${\beta }_{{X}_{6}}$	1

An individual’s outcome measure was defined to be dependent on the main effects of $T$, $M$ and ${X}_{1}, \dots ,{X}_{6}$, as well as a product term between $T$ and $M$ (representing the treatment effect modification effect) and a product term between $M$ and ${X}_{1}$ representing modification of the effect of ${X}_{1}$ on the outcome by $M$:

$$Y\sim N\left({\beta }_{0}+{\beta }_{T}T+{\beta }_{M}M+{\beta }_{TM}TM+{\beta }_{M{X}_{1}}M{X}_{1}+\sum_{k=1}^{6}{\beta }_{{X}_{j}}{X}_{j}, 0.2\right)$$

The chosen values of the $\beta$ coefficients are given in Table 1. These were not based on a specific dataset but agreed as realistic values for an observational study.

The simulations considered three different values of the coefficients ${\alpha }_{M{X}_{1}}$ and ${\beta }_{M{X}_{1}}$ corresponding to null, moderate and large effect sizes of the $M\times {X}_{1}$ term on treatment receipt and the $M\times {X}_{1}$ term on outcome respectively (Table 1). Two values for ${\beta }_{TM}$ were chosen to represent small and moderate treatment effect modification effect respectively (Table 1). All other coefficient values were kept fixed. This resulted in 18 different combinations of modification effect sizes.

#### Confounding adjustment methods

Four methods of confounding adjustment were considered: (1) regression adjustment, (2) PS covariate adjustment (where the estimated PS score is added as a linear term to the outcome model), (3) inverse probability of treatment weighting (IPTW) using the propensity score, and (4) PS nearest neighbour one-to-one matching, with replacement. For each, four confounding adjustment models were considered, the first three being (a) adjusting for the main effects of$M$, ${X}_{1},\dots ,{X}_{6}$, but no product terms, (b) adjusting for the main effects of$M$, ${X}_{1},\dots ,{X}_{6}$ and an $M\times {X}_{1}$ product term, and (c) adjusting for the main effects of$M$, ${X}_{1},\dots ,{X}_{6}$ and six product terms${X}_{1}\times M, {X}_{2}\times M,\dots , {X}_{6}\times M$. The fourth model (d) was the adjustment of confounding separately within each subgroup of$M$, thus separate linear models were fit in the two subgroups. This involved estimating subgroup-specific PS models. Here, the term ‘adjustment model’ refers to the propensity score for the three propensity score methods and the outcome model for regression adjustment.

The estimated individual propensity scores were obtained by fitting a logistic regression model, regressing treatment receipt on the set of confounders, including $M$. The individual inverse probability of treatment weights were defined as the inverse of the probability of that individual receiving the treatment allocation they did receive. The nearest neighbour matching was performed with no specified calliper.

For each confounding model and method combination (16 in total), estimates of the subgroup-specific treatment effects, ${\widehat{\beta }}_{T|M=1}$ and ${\widehat{\beta }}_{T| M=0}$, and the treatment-effect moderation estimate, ${\widehat{\beta }}_{TM}= {\widehat{\beta }}_{T|M=1}- {\widehat{\beta }}_{T| M=0}$ were obtained via a linear regression model.

#### Parameter estimation

500 simulations were run per scenario (18 combinations of moderation effect sizes). This number of simulations was determined to be a conservative number required to detect a treatment-moderator interaction effect size of 0.3 within an accuracy of 10% when the sample size was 1000 [14]. For each scenario, the mean of the 500 estimates of ${\widehat{\beta }}_{T|M=1}$, ${\widehat{\beta }}_{T|M=0}$ and ${\widehat{\beta }}_{TM}$ from the 16 confounding adjustment method/model combinations were obtained, along with the empirical standard error (calculated as the standard deviation of the estimates over the 500 simulations) and the average model standard error [15].

### Applied example

To demonstrate the potential impact of accounting for interactions between a moderator and a confounder in practice, we used a dataset comprising information from the 2018–19 National Survey for Wales, a large-scale cross-sectional survey run annually by the Office for National Statistics on behalf of the Welsh Government. Participants are randomly selected from the population of Wales and asked a variety of questions regarding their health, lifestyle and interests. The anonymised data is available from the UK Data Service [16].

The specific example relates to the estimation of experiencing tinnitus on mental well-being. Tinnitus is a self-reported binary measure (experiences tinnitus or does not experience tinnitus) and mental well-being was measured using the Warwick-Edinburgh Mental Well-being Scale, a numerical scale scored between 14–70 where a higher score indicates a higher level of mental well-being. We investigated whether the effect of tinnitus on mental well-being was moderated by three binary variables: gender, ethnicity (White/non-White) or current smoking status (currently smoke/currently do not smoke). Additional potential confounders accounted for were age (in years) and BMI (as a numerical variable).

A series of linear regression models were fitted including interactions between tinnitus and each of the three potential moderators. Confounding was adjusted for via both regression adjustment and IPTW. In the assessment of each of the three moderators, the other two potential moderators were included as potential confounders. Two confounding adjustment models for each method were specified, the first adjusting only for the main effects of each confounder, the second adjusting for the main effects of each confounder and interactions between the moderator and each confounder.

### Software

The simulation study and analysis for the applied example were performed in Stata version 14 [17].

## Results

### Simulation study

**Fig. 2:**
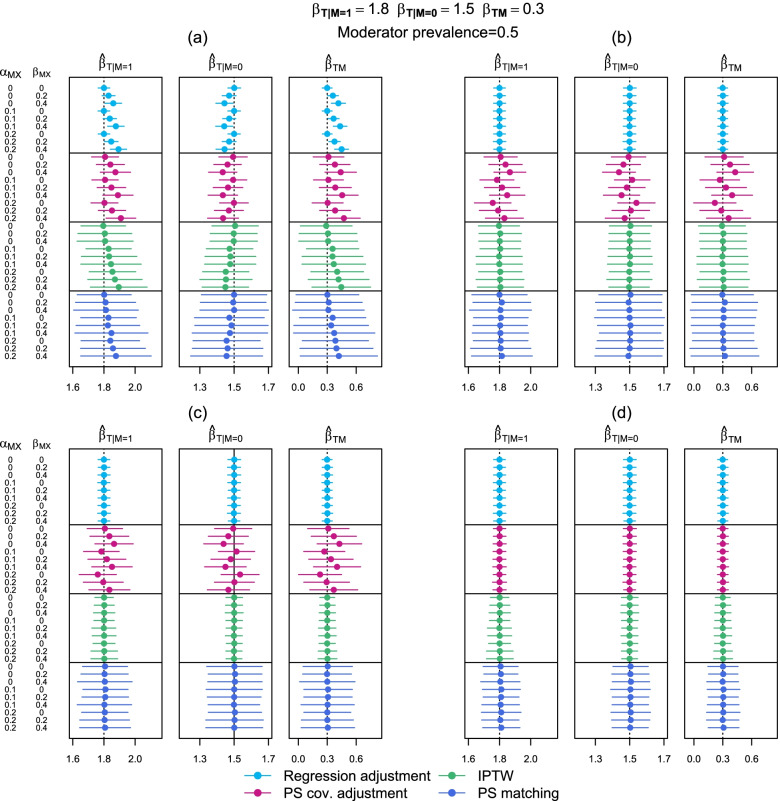
Estimated subgroup-specific treatment effects and effect modification where the moderator has a prevalence of 0.5. ${\widehat{\beta }}_{T|M=m}$ is the subgroup-specific treatment effect and ${\widehat{\beta }}_{TM}$ is the treatment effect moderator effect. (**a**) adjusting for no moderator-confounders interactions, (**b**) adjusting for the one moderator-confounder interaction, (**c**) adjusting for all possible moderator-confounder interactions, (**d**) subgroup-specific confounding adjustment. (1) Regression adjustment, (2) Propensity score covariate adjustment, (3) IPTW, (4) Propensity score matching

**Fig. 3:**
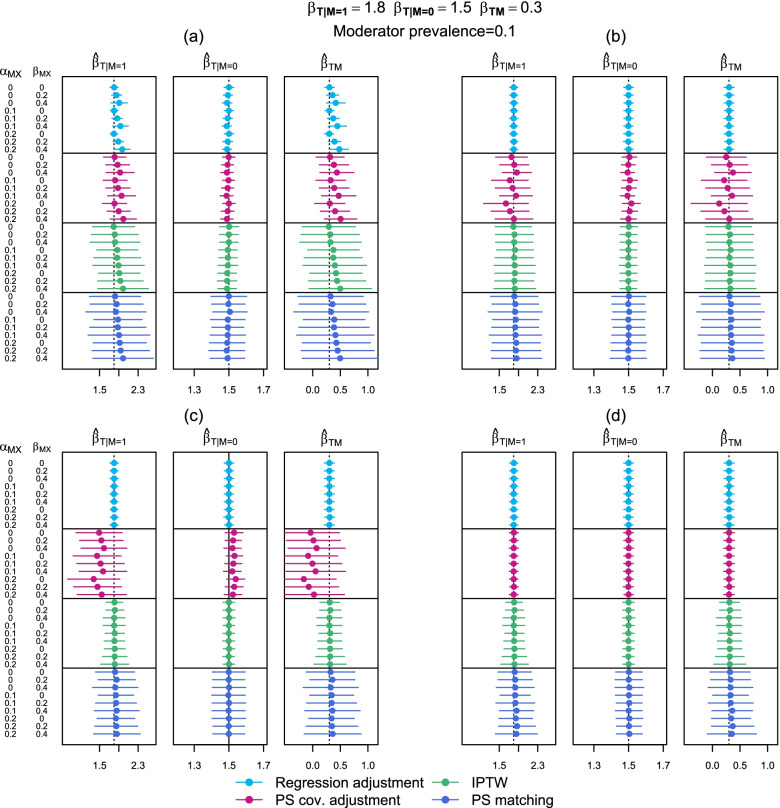
Estimated subgroup-specific treatment effects and effect modification where the moderator has a prevalence of 0.1. ${\widehat{\beta }}_{T|M=m}$ is the subgroup-specific treatment effect and ${\widehat{\beta }}_{TM}$ is the treatment effect moderator effect. (**a**) adjusting for no moderator-confounders interactions, (**b**) adjusting for the one moderator-confounder interaction, (**c**) adjusting for all possible moderator-confounder interactions, (**d**) subgroup-specific confounding adjustment. (1) Regression adjustment, (2) Propensity score covariate adjustment, (3) IPTW, (4) Propensity score matching

The forest plots in Figs. 2 and 3 show the estimated values of ${\beta }_{T|M=1}$, ${\beta }_{T|M=0}$ and ${\beta }_{TM}$ for the various scenarios regarding the magnitude of the $M\times {X}_{1}$ effect size on both treatment receipt and outcome and for each of the confounding adjustment methods and models tested, averaged over the 500 simulations, where ${\beta }_{T|M=1}=1.8$, ${\beta }_{T|M=0}=1.5$ and ${\beta }_{TM}=0.3$. The 95% confidence intervals (obtained using the estimated standard error assuming a normal distribution) are displayed. The prevalence $M$ was 0.5 for the results in Fig. 2 and 0.1 for Fig. 3. Tables displaying these results are given the Supplementary material.

We first discuss the patterns of bias observed when no moderator-confounder interactions were accounted for in the confounding adjustment (i.e. confounding model (a)). In Fig. 2 a non-zero ${\alpha }_{M{X}_{1}}$ did not introduce bias into either the subgroup-specific treatment effect estimates or the interaction effect estimate ${\widehat{\beta }}_{TM}$ when the confounding method was regression adjustment or PS covariate adjustment. However, there was bias in estimates obtained from these two methods when ${\beta }_{M{X}_{1}}>0$. Alternatively, for IPTW and PS matching, increasing ${\alpha }_{M{X}_{1}}$ from 0 did induce bias into the subgroup and the interaction effect. Increasing ${\beta }_{M{X}_{1}}$ also appeared to have a slight impact on the estimates from these latter two confounding adjustment methods. For all confounding adjustment methods, the magnitude of the bias increased as the effect size of the relevant moderator-confounder interactions increased.

In this simulation study, the direction of bias was always positive for ${\widehat{\beta }}_{T|M=1}$ and the interaction effect, ${\widehat{\beta }}_{TM}$, and negative for ${\widehat{\beta }}_{T|M=0}$ as the impact of the confounder ${X}_{1}$ on both treatment receipt and the outcome was set to be larger when $M=1$ (since both ${\alpha }_{M{X}_{1}}>0$ and ${\beta }_{M{X}_{1}}>0$). When the average effect of ${X}_{1}$ was adjusted for equally in both groups of $M$, this led to an under-adjustment of ${X}_{1}$ when $M=1$ and an over-adjustment of ${X}_{1}$ when $M=0$. Because the inclusion of ${X}_{1}$ attenuated the estimated treatment effect, this led to an overestimation of the treatment effect when $M=1$ and an underestimation when $M=0$.

When an $M\times {X}_{1}$ interaction term was included in the confounding adjustment model, i.e. for confounding model (b), the bias in each three parameter estimates was substantially reduced for regression adjustment, IPTW and PS matching. For PS covariate adjustment, a similar pattern of bias was seen as for confounding adjustment model (a).

Including all possible moderator-confounder interactions in the confounding adjustment model, i.e. adjustment model (c), and performing the stratified analysis, i.e. adjustment model (d), also resulted in substantially reduced levels of bias in the subgroup-specific treatment effects and ${\widehat{\beta }}_{TM}$ for regression adjustment, IPTW and PS matching. Confounding models (c) and (d) gave the exact same results for regression adjustment and IPTW due to their nature. Adjustment model (c) resulted in similar biased estimates to (b) for PS covariate adjustment. Only in the stratified analysis did PS covariate adjustment produce accurate estimates.

In Fig. 3, the magnitude of bias in the ${\widehat{\beta }}_{T|M=1}$ subgroup treatment effect was larger than in Fig. 2 and the magnitude of bias in the ${\widehat{\beta }}_{T|M=0}$ was smaller. The bias in the overall interaction effect was similar but overall the confidence intervals were wider in Fig. 3. Another discrepancy between Figs. 2 and 3 is seen for PS covariate adjustment using IPTW (model (c), method (2)). Even where ${\alpha }_{M{X}_{1}}=0$ and ${\beta }_{M{X}_{1}}=0$, all estimates were biased when the prevalence of the moderator was 0.1. Otherwise, Figs. 2 and 3 showed similar patterns of bias.

When ${\beta }_{T|M=1}$, ${\beta }_{T|M=0}$ and ${\beta }_{TM}$ were larger, the magnitude of bias was the same but the impact of the bias in the TEM estimate is smaller relative to its larger magnitude (supplementary tables 1, 2, 3, 4, 5, 6, 7, 8).

To more thoroughly compare the accuracy in estimates across all models for each adjustment method, we compared the mean bias in ${\widehat{\beta }}_{TM}$ across the set of ${\alpha }_{M{X}_{1}}$ and ${\beta }_{M{X}_{1}}$ values (Table 2).

**Table 2:** Average absolute bias $\left|{\widehat{\beta }}_{TM}-{\beta }_{TM}\right|$ for the different confounding adjustment methods for confounding adjustment models (b)-(d)

		**Adjustment model**			
	**Adjustment method**	**(a)**	**(b)**	**(c)**	**(d)**
${{\varvec{\beta}}}_{{\varvec{T}}{\varvec{M}}}=0.3$ **Prev.**${\varvec{M}}=0.5$	**(1)**	0.066664	0.000773	0.000791	0.000791
	**(2)**	0.082535	0.059600	0.058446	0.000765
	**(3)**	0.063750	0.005581	0.001400	0.001400
	**(4)**	0.055594	0.008806	0.004108	0.004226
${{\varvec{\beta}}}_{{\varvec{T}}{\varvec{M}}}=0.3$ **Prev.**${\varvec{M}}=0.1$	**(1)**	0.075085	0.001873	0.001715	0.001715
	**(2)**	0.093259	0.062743	0.322948	0.001586
	**(3)**	0.083261	0.017981	0.011239	0.011239
	**(4)**	0.096792	0.038555	0.043617	0.038939
${{\varvec{\beta}}}_{{\varvec{T}}{\varvec{M}}}=0.6$ **Prev.**${\varvec{M}}=0.5$	**(1)**	0.066454	0.000816	0.000749	0.000749
	**(2)**	0.081596	0.059213	0.056933	0.000941
	**(3)**	0.060059	0.003119	0.002269	0.002269
	**(4)**	0.052857	0.006547	0.007896	0.005279
${{\varvec{\beta}}}_{{\varvec{T}}{\varvec{M}}}=0.6$ **Prev.**${\varvec{M}}=0.1$	**(1)**	0.073871	0.000848	0.000925	0.000925
	**(2)**	0.092722	0.056906	0.319295	0.00106
	**(3)**	0.074819	0.013062	0.012955	0.012955
	**(4)**	0.079721	0.036695	0.046072	0.041207

Confounding models: (a) adjusting for no moderator-confounder interactions, (b) adjusting for the one moderator-confounder interaction, (c) adjusting for all possible moderator-confounder interactions, (d) subgroup-specific confounding adjustment

Confounding methods: (1) Regression adjustment, (2) Propensity score covariate adjustment, (3) IPTW, (4) Propensity score matching

As expected, the average bias tended to be highest when no moderator-confounder interactions were adjusted for (adjustment model (a)). Overall, the average bias was still reasonably small in magnitude for adjustment model (a); however, this is the average over all ${\alpha }_{M{X}_{1}}$ and ${\beta }_{M{X}_{1}}$ values, and the bias was larger (up to 0.15 in magnitude) as ${\alpha }_{M{X}_{1}}$ and ${\beta }_{M{X}_{1}}$ increased.

The average bias in the estimation of ${\widehat{\beta }}_{TM}$ was the same (to 4dp) across adjustment models (b)-(d) for regression adjustment. For IPTW, the bias was smaller when all moderator-confounder interactions were accounted for and when a stratified analysis was performed than when only the one moderator-confounder was accounted for, and more so when the prevalence of M was 0.5. There was not a clear pattern for PS matching.

The precision of the various estimates of ${\widehat{\beta }}_{T}$ and ${\widehat{\beta }}_{TM}$, i.e. how much confidence we have that sample estimates reflect the population parameter, can be most easily assessed in Figs. 2 and 3 and the results tables in the supplementary material by examining the width of the 95% confidence intervals. Confounding models (b), (c) and (d) gave similar levels of precision for estimates obtained via regression adjustment. For PS covariate adjustment, the precision (as well as the accuracy) of estimates was highest for confounding model (d), i.e. in the stratified analysis. For IPTW, the precision was higher for confounding models (c) and (d) than confounding models (a) and (b). For PS matching, the precision was highest for confounding model (d).

Comparing the empirical and average model standard error is a way of assessing bias in the estimation of the model standard error [15]. For regression adjustment, the average model standard errors are very close to the empirical standard errors across all models and scenarios, suggesting this methods accurately estimate the standard errors of the estimates. However, for the other methods using propensity scores, particularly IPTW and PS matching, the average model standard errors typically overestimated the empirical standard error, sometimes severely. The difference between the two standard errors was largest for confounding model d.

Morris et al. say that the comparison of the empirical standard error and the average model standard error should be interpreted with caution when the methods are known to be biased as the empirical SEs can be small as a result [15]. However, large differences were seen even when the method was not biased in the estimates of ${\widehat{\beta }}_{T}$ and ${\widehat{\beta }}_{TM}$. Other studies have shown that when propensity scores are used, the average model standard error can be larger than the empirical standard error [18, 19]. We suspect this is due to the use of the robust variance estimator in these models as this can overestimate the variance of effect to protect against some element of misspecification [19].

This analysis did not primarily seek to compare the accuracy and precision of the different confounding adjustment methods overall. In general, estimates obtained via regression adjustment had the smallest bias. However, this is likely to reflect the way in which the data was simulated, and will likely not be true in all applications. Estimates obtained via IPTW had noticeably higher precision than PS matching, but it is possible that a more sophisticated version of PS matching would have performed better.

### Applied example

Table 3 displays the interaction effect estimates for tinnitus and each of gender, white ethnicity, and current smoking status on mental well-being obtained from fitting a series of linear regression models. Confounding was adjusted for (separately) via both regression adjustment and IPTW, and the confounding adjustment models included either no moderator-confounder interactions or all possible moderator-confounder interactions. The sample size in all models was 5402 observations.

**Table 3:** The interaction effect estimates between tinnitus and several additional variables. Confounding was adjusted for via both regression adjustment and IPTW, firstly when no moderator-confounder interactions were accounted for in the adjustment model and secondly when all possible moderator-confounder interactions were accounted for in the adjustment model

	**Regression adjustment**		**IPTW**	
	**No **${\varvec{M}}\times {\varvec{X}}$** interactions**	**All **${\varvec{M}}\times {\varvec{X}}$** interactions**	**No **${\varvec{M}}\times {\varvec{X}}$** interactions**	**All **${\varvec{M}}\times {\varvec{X}}$** interactions**
	**Estimate** **(95% CI)**	**Estimate** **(95% CI)**	**Estimate** **(95% CI)**	**Estimate** **(95% CI)**
**Gender, female**	2.56 (-0.80, 5.92)	2.86 (-0.52, 6.23)	2.60 (-0.84, 6.04)	2.37 (-1.07, 5.82)
**White ethnicity**	-0.92 (-10.13, 8.28)	-2.84 (-12.10, 6.43)	-2.84 (-12.97, 7.29)	-4.24 (-15.06, 6.57)
**Current smoking**	-3.72 (-7.95, 0.50)	-3.93 (-8.17, 0.31)	-3.62 (-7.86, 0.62)	-3.48 (-7.70, 0.74)

Adjusting for interactions between the moderator and confounders in regression adjustment had little difference on the estimates of interaction between tinnitus and each of gender and current smoking status. Although not statistically significant in either case, the interaction effect between tinnitus and white ethnicity roughly tripled in magnitude when moderator-confounder interactions were adjusted for. Further inspection showed that an interaction between White ethnicity and age was present and statistically significant. This implies that the effect of age on mental well-being onset was different for people of White and non-White ethnicity.

Again, when IPTW was applied, adjusting for interactions between the moderator and confounders in the propensity score model had little difference on the estimates of interaction between tinnitus and each of gender and current smoking status. interaction effect between tinnitus and White ethnicity increased in magnitude when moderator-confounder interactions were included in the propensity score model. Upon further inspection, none of the interactions between White ethnicity and the confounders were of notable size or were statistically significant, however, the combined effect of their inclusion still had an impact on the overall interaction effect between White ethnicity and tinnitus on mental well-being.

We did not aim to provide a robust answer to the clinical question posed as there are limitations regarding unmeasured confounding and the dichotomisation of smoking status and ethnicity. However, this practical application shows that different point estimates of effect modification may be obtained in practice depending on whether interactions between the moderator of interest and the confounders are included in the confounding adjustment. In many cases, the differences may be marginal and the overall conclusions will not change. In some cases however, the differences may lead to different conclusions being made.

## Discussion

### Summary of findings

Our findings confirm that failure to account for any interactions present between the moderator and a confounder on treatment receipt introduced bias into subgroup-specific and TEM estimates when IPTW and PS matching was applied [8–11, 12]. Our simulations also indicated that the presence of moderator-confounder interactions on the outcome induced a small amount of bias into parameter estimates. Both adjusting for the relevant (or all possible) moderator-confounder interactions in the propensity score creation and estimating subgroup-specific PS models removed this bias.

Whilst it is not surprising, to our knowledge it has not been previously clarified that PS covariate adjustment is instead sensitive to failure to account for interactions between the moderator and a confounder on outcome. Hence, inclusion of confounder-moderator interactions in the PS model does not rectify this problem; only when subgroup-specific PS models were estimated did PS covariate adjustment produce accurate estimates. Similarly, regression adjustment produced biased estimates where there existed a moderator-confounder interaction on outcome which was not accounted for.

The accuracy and precision of estimates (based on the empirical standard errors) obtained from regression adjustment were similar when only the one moderator-confounder interaction was accounted for in the confounding model, when all possible moderator-confounder interactions were accounted for and when the stratified analysis was performed. For IPTW, the accuracy and precision was higher when all possible moderator-confounder interactions were accounted for and when the stratified analysis was performed, compared to when just the one moderator-confounder interaction was accounted for. For PS matching, the accuracy and precision was highest in the stratified analysis. However, in the methods using propensity scores, particularly IPTW and PS matching, the average model standard errors tended to overestimate the empirical standard errors which would lead to less precision of the subgroup and moderator effect estimates in practice when these methods were used.

If the moderator itself is a confounder, by not accounting for any moderator-confounder interactions that exist on either treatment or outcome, one is essentially misspecifying the propensity score or outcome model. This should in theory induce bias into any estimates obtained from the outcome model and it is already recommended that confounder-confounder interactions be considered [20] although this is not always done. However, it seems plausible that when the interest is specifically in treatment effect modification, not accounting for existing moderator-confounder interactions will have a more serious impact on accuracy than not accounting for other confounder-confounder interactions. Furthermore, the magnitude of treatment effect modification is typically small relative to the magnitude of main effects, thus such estimates may be more sensitive to bias.

The applied example demonstrated the potential impact of accounting for moderator-confounder interactions. In many cases, the difference in the estimates of treatment effect modification obtained when moderator-confounder interactions were and were not accounted for was very small. However, a difference was observed for some.

Although we did not include these in our simulation studies, doubly robust estimators are an attractive way of estimating the effect of exposures on outcomes in observational studies [21]. Doubly robust estimators use both the outcome model and propensity score, giving an unbiased effect estimate if at least one is correctly specified. Hence, if interactions exist between the moderator and one or more confounders on either treatment receipt or the outcome, but not both, and these are not accounted for, doubly robust estimation should still provide unbiased estimates. However, it is still advisable to consider the presence of interactions between a potential moderator and the confounders on both treatment receipt and the outcome, to avoid potential misspecification of both the outcome model and propensity score.

### Limitations

In this study, we considered four different methods of adjusting for confounding. Other methods which could have been considered include stratification by the PS and other versions of PS matching. The aim of this analysis was not however to compare the different methods in terms of accuracy and precision, but to explore the bias within each method. We suspect that, in general, methods based on the PS will always be prone to bias when there are interactions between the moderator and confounder on treatment assignment (the exception being PS covariate adjustment).

In the simulations, we only considered situations in which there was a linear interaction between the moderator and confounder when either was continuous. In practice, there may be more complex non-linear interactions between the moderator and confounder which may be insufficiently accounted for with a linear interaction term in the confounding adjustment. If this is the case, this should be incorporated into the confounding adjustment if possible.

Additionally, we only considered scenarios with a continuous outcome and a binary moderator. We expect similar patterns of bias to be seen with other outcome and moderator types, but the relative accuracy and precision of the confounding adjustment models within each method may not be the same. We also only simulated data where there were six confounders and only one moderator of interest, but in practice there may be many more confounders and moderators of interest. It may be that a moderator interacts with multiple confounders and, if the bias introduced by each moderator-confounder pair were in the same direction, the overall amount of bias on the estimation of treatment effect modification could be substantial. Alternatively, the different biases could cancel each other out if they acted in different directions.

It has been recommended that a propensity score model include not only confounders, but also variables associated with the outcome as this increases precision [22]. It seems intuitive that interactions between the moderator(s) and variables only associated with the outcome do not need to be considered in a propensity score model, as the moderator cannot influence the effect of such variables on treatment receipt if the variable does not have an effect on treatment receipt.

Here, we consider a simplistic, although common, approach to assessing treatment effect modification as only one moderator is considered at a time in a parametric model. More sophisticated and flexible approaches exist which allow researchers to assess treatment heterogeneity more generally. Bayesian additive regression trees (BART), for example, avoid the strong parametric assumptions required for the standard linear and logistic regression models and automates the detection of interactions [23]. Additionally, the Bayesian causal forest model works particularly well when there is strong confounding [24]. Many of these methods are readily available in R. For example, the EffectLiteR package in R enables the estimation of average and conditional effects whilst taking into account any number of continuous and categorical covariates, can estimate multiple interaction effects simultaneously [25].

## Conclusion

In conclusion, we recommend that the presence of moderator-confounder interactions are considered and accounted for when estimating treatment effect medication whilst adjusting for additional variables. Accounting for moderator-confounder interactions that did not exist did not have a negative impact in our simulation study, hence we suggest that researchers include interactions terms if they are undecided about their presence. However, this approach may be unattractive when using regression adjustment with a smaller sample size. We also recommend that subgroup-specific propensity scores are created and used in a stratified analysis when using propensity score covariate adjustment to assess treatment effect modification by a binary variable.

## Supplementary Information

**Additional file 1. **

## Data Availability

Stata code and the simulated data will be shared upon request via Antonia Marsden.

## Abbreviations

TEM: Treatment effect modification
PS: Propensity score
IPTW: Inverse probability of treatment weighting
