# Supplementary material for: The impact of moderator by confounder interactions in the assessment of treatment effect modification: a simulation study
Source: BMC Med Res Methodol. 2022 Apr 3;22:88. doi: 10.1186/s12874-022-01519-7 (PMC8978434; doi:10.1186/s12874-022-01519-7)
Supplement: Supplementary file 1 — Additional file 1. [file 12874_2022_1519_MOESM1_ESM.docx]

**The impact of moderator by confounder interactions in the assessment of treatment effect modification: a simulation study
Supplementary material**

# Description of potential biases

Assume estimation of treatment effect modification by a binary variable $M$ on the relationship between a binary treatment $T$ on some outcome $Y$ in the presence of a confounder $X$ is of interest. Failure to adjust for $X$ will induce bias into the treatment effect estimates within the subgroups of $M$ due to unmeasured confounding. If $X$ is accounted for but insufficiently so, e.g. from crude categorisation, this will induce bias from residual confounding.

Suppose that the effect of $X$ on $Y$ is larger in one subgroup of $M$ than the other. If only the main effect of $X$ is accounted for in the adjustment, i.e. if the effect of $X$ on $Y$ is assumed to be constant over $M$, this will result in an overestimation of the effect of $X$ on $Y$ in one subgroup of $M$, and an underestimation in the other. Therefore there will be bias acting in the opposite direction in the subgroup-specific treatment effect estimates due to residual confounding.

Regression adjustment controls for confounding by conditioning on the relationship between the confounders and the outcome, i.e. removing the line between $X$ and $Y$ in Figure 1. Therefore, it would be expected that that this method will be prone to bias when the moderator influences the effects of confounder on outcome. Alternatively, propensity score methods aim to control for confounding by modelling the relationship between the confounders and treatment receipt, i.e. removing the line between $X$ and $T$ in Figure 1. Therefore, it would be expected that this method will be prone to bias when the moderator influences the effects of confounder on treatment receipt. Propensity score covariate adjustment is different from other PS methods in that it models the relationship between the covariates and treatment receipt, but confounding is controlled for by conditioning on the relationship between the propensity score and the outcome.

The magnitude and the impact of bias due to unaccounted modification of the confounder effects on treatment receipt and/or the outcome will depend on 1) the prevalence of $M$ (if $M$ is binary), 2) the relative effect sizes of the modification of the treatment effect and modification of the confounder effects, and 3) the number of confounders whose effects are influenced by the moderator.

1. *The prevalence of* $M$

Suppose the effect of $X$ on $Y$ in the $M=1$subgroup is $\beta_{X, M=1}$ and the effect of $X$ on $Y$ in the $M=0$ subgroup is $\beta_{X, M=0}$. If the prevalence of $M$ is 0.5 and we adjust for a constant effect of $X$ on $Y$ across subgroups of $M$ in a linear model, the average effect of $X$ on $Y$ is $\beta_{X}=0.5\times\beta_{X, M=1}+0.5\times\beta_{X, M=0}$ which is equidistant to $\beta_{X, M=1}$ and $\beta_{X, M=0}$ and should result in an equal absolute amount of bias in the subgroups of $M$. If the prevalence of $M$ is 0.1, the average effect of $X$and $Y$ across subgroups is $\beta_{X}=0.1\times\beta_{X, M=1}+0.9\times\beta_{X, M=0}$ which is closer to $\beta_{X, M=0}$ thus resulting in larger bias in subgroup $M=1$ compared to $M=0$. However, this balance of the bias between the subgroups of $M$ should not impact the overall bias in the TEM estimate.

1. *The relative effect sizes of the modification of the treatment effect and modification of the confounder effects*

If the TEM effect that we are aiming to estimate is small, but the modification of the confounder effect is quite large, the TEM effect will potentially be very biased. On the other hand, if the TEM effect that we are aiming to estimate is large, but the modification of the confounder effect is quite small, failure to account for modification of the confounder effect may only introduce negligible bias into the TEM effect estimate.

1. *The number of confounders whose effects are influenced by the moderator*

In practice, the moderator may influence the effects of several confounders. The overall bias in the estimate of treatment effect modification arising from failure to account for these will be a combination of the individual biases from each confounder. Depending on the magnitude and direction of each of these individual biases, there may be little overall bias if the individual biases cancel each other out, or a large degree of bias if they act in the same direction.

# Simulation study results tables

Table S1: Mean (M) and standard deviation (SD) of subgroup-specific treatment effects ($\hat{\beta}_{T|M=m}$) and treatment effect modification ($\hat{\beta}_{TM}$) estimated via regression adjustment from the 500 simulated datasets where the moderator had a prevalence of 0.5

|  |  |  | **Confounding model a** | | | | | | | | | |
| --- | --- | --- | --- | --- | --- | --- | --- | --- | --- | --- | --- | --- |
|  |  |  | ${\hat{\boldsymbol{\beta}}}_{\boldsymbol{T\vert M=0}}$ | | | ${\hat{\boldsymbol{\beta}}}_{\boldsymbol{T\vert M=1}}$ | | | ${\hat{\boldsymbol{\beta}}}_{\boldsymbol{TM}}$ | | | |
| $\boldsymbol{\beta}_{\boldsymbol{TM}}$**^2^** | $\boldsymbol{\alpha}_{\boldsymbol{M}\boldsymbol{X}_{\boldsymbol{1}}}$**^3^** | $\boldsymbol{\beta}_{\boldsymbol{M}\boldsymbol{X}_{\boldsymbol{1}}}$**^4^** | **M** | $\mathbf{SE}_{\mathbf{E}}$ | $\mathbf{SE}_{\mathbf{M}}$ | **M** | $\mathbf{SE}_{\mathbf{E}}$ | $\mathbf{SE}_{\mathbf{M}}$ | **M** | $\mathbf{SE}_{\mathbf{E}}$ | $\mathbf{SE}_{\mathbf{M}}$ | **p^5^** |
| **0.3** | **0** | **0** | 1.50 | 0.02 | 0.02 | 1.80 | 0.02 | 0.02 | 0.30 | 0.03 | 0.03 | 0.390 |
| **0.3** | **0** | **0.2** | 1.47 | 0.02 | 0.02 | 1.83 | 0.02 | 0.02 | 0.36 | 0.03 | 0.03 | <0.001 |
| **0.3** | **0** | **0.4** | 1.44 | 0.03 | 0.03 | 1.86 | 0.03 | 0.03 | 0.42 | 0.04 | 0.04 | <0.001 |
| **0.3** | **0.1** | **0** | 1.50 | 0.02 | 0.02 | 1.80 | 0.02 | 0.02 | 0.30 | 0.03 | 0.03 | 0.999 |
| **0.3** | **0.1** | **0.2** | 1.47 | 0.02 | 0.02 | 1.84 | 0.02 | 0.02 | 0.37 | 0.03 | 0.03 | <0.001 |
| **0.3** | **0.1** | **0.4** | 1.44 | 0.03 | 0.03 | 1.88 | 0.03 | 0.03 | 0.43 | 0.04 | 0.04 | <0.001 |
| **0.3** | **0.2** | **0** | 1.50 | 0.02 | 0.02 | 1.80 | 0.02 | 0.02 | 0.30 | 0.03 | 0.03 | 0.999 |
| **0.3** | **0.2** | **0.2** | 1.47 | 0.02 | 0.02 | 1.85 | 0.02 | 0.02 | 0.38 | 0.03 | 0.03 | <0.001 |
| **0.3** | **0.2** | **0.4** | 1.44 | 0.03 | 0.03 | 1.89 | 0.03 | 0.03 | 0.45 | 0.04 | 0.04 | <0.001 |
| **0.6** | **0** | **0** | 1.50 | 0.02 | 0.02 | 2.10 | 0.02 | 0.02 | 0.60 | 0.03 | 0.03 | 0.098 |
| **0.6** | **0** | **0.2** | 1.47 | 0.02 | 0.02 | 2.13 | 0.02 | 0.02 | 0.66 | 0.03 | 0.03 | <0.001 |
| **0.6** | **0** | **0.4** | 1.44 | 0.03 | 0.03 | 2.16 | 0.03 | 0.03 | 0.71 | 0.04 | 0.04 | <0.001 |
| **0.6** | **0.1** | **0** | 1.50 | 0.02 | 0.02 | 2.10 | 0.02 | 0.02 | 0.60 | 0.03 | 0.03 | 0.999 |
| **0.6** | **0.1** | **0.2** | 1.47 | 0.02 | 0.02 | 2.14 | 0.02 | 0.02 | 0.67 | 0.03 | 0.03 | <0.001 |
| **0.6** | **0.1** | **0.4** | 1.44 | 0.03 | 0.03 | 2.18 | 0.03 | 0.03 | 0.73 | 0.03 | 0.04 | <0.001 |
| **0.6** | **0.2** | **0** | 1.50 | 0.02 | 0.02 | 2.10 | 0.02 | 0.02 | 0.60 | 0.02 | 0.03 | 0.352 |
| **0.6** | **0.2** | **0.2** | 1.47 | 0.02 | 0.02 | 2.15 | 0.02 | 0.02 | 0.68 | 0.03 | 0.03 | <0.001 |
| **0.6** | **0.2** | **0.4** | 1.44 | 0.03 | 0.03 | 2.20 | 0.03 | 0.03 | 0.75 | 0.04 | 0.04 | <0.001 |
|  |  |  | **Confounding model b** | | | | | | | | | |
|  |  |  | ${\hat{\boldsymbol{\beta}}}_{\boldsymbol{T\vert M=0}}$ | | | ${\hat{\boldsymbol{\beta}}}_{\boldsymbol{T\vert M=1}}$ | | | ${\hat{\boldsymbol{\beta}}}_{\boldsymbol{TM}}$ | | | |
| $\boldsymbol{\beta}_{\boldsymbol{TM}}$**^2^** | $\boldsymbol{\alpha}_{\boldsymbol{M}\boldsymbol{X}_{\boldsymbol{1}}}$**^3^** | $\boldsymbol{\beta}_{\boldsymbol{M}\boldsymbol{X}_{\boldsymbol{1}}}$**^4^** | **M** | $\mathbf{SE}_{\mathbf{E}}$ | $\mathbf{SE}_{\mathbf{M}}$ | **M** | $\mathbf{SE}_{\mathbf{E}}$ | $\mathbf{SE}_{\mathbf{M}}$ | **M** | $\mathbf{SE}_{\mathbf{E}}$ | $\mathbf{SE}_{\mathbf{M}}$ | **p^5^** |
| **0.3** | **0** | **0** | 1.50 | 0.02 | 0.02 | 1.80 | 0.02 | 0.02 | 0.30 | 0.03 | 0.03 | 0.390 |
| **0.3** | **0** | **0.2** | 1.50 | 0.02 | 0.02 | 1.80 | 0.02 | 0.02 | 0.30 | 0.03 | 0.03 | 0.408 |
| **0.3** | **0** | **0.4** | 1.50 | 0.02 | 0.02 | 1.80 | 0.02 | 0.02 | 0.30 | 0.03 | 0.03 | 0.999 |
| **0.3** | **0.1** | **0** | 1.50 | 0.02 | 0.02 | 1.80 | 0.02 | 0.02 | 0.30 | 0.03 | 0.03 | 0.408 |
| **0.3** | **0.1** | **0.2** | 1.50 | 0.02 | 0.02 | 1.80 | 0.02 | 0.02 | 0.30 | 0.03 | 0.03 | 0.371 |
| **0.3** | **0.1** | **0.4** | 1.50 | 0.02 | 0.02 | 1.80 | 0.02 | 0.02 | 0.30 | 0.03 | 0.03 | 0.408 |
| **0.3** | **0.2** | **0** | 1.50 | 0.02 | 0.02 | 1.80 | 0.02 | 0.02 | 0.30 | 0.03 | 0.03 | 0.999 |
| **0.3** | **0.2** | **0.2** | 1.50 | 0.02 | 0.02 | 1.80 | 0.02 | 0.02 | 0.30 | 0.03 | 0.03 | 0.098 |
| **0.3** | **0.2** | **0.4** | 1.50 | 0.02 | 0.02 | 1.80 | 0.02 | 0.02 | 0.30 | 0.03 | 0.03 | 0.999 |
| **0.6** | **0** | **0** | 1.50 | 0.02 | 0.02 | 2.10 | 0.02 | 0.02 | 0.60 | 0.03 | 0.03 | 0.098 |
| **0.6** | **0** | **0.2** | 1.50 | 0.02 | 0.02 | 2.10 | 0.02 | 0.02 | 0.60 | 0.03 | 0.03 | 0.999 |
| **0.6** | **0** | **0.4** | 1.50 | 0.02 | 0.02 | 2.10 | 0.02 | 0.02 | 0.60 | 0.03 | 0.03 | 0.372 |
| **0.6** | **0.1** | **0** | 1.50 | 0.02 | 0.02 | 2.10 | 0.02 | 0.02 | 0.60 | 0.03 | 0.03 | 0.999 |
| **0.6** | **0.1** | **0.2** | 1.50 | 0.02 | 0.02 | 2.10 | 0.02 | 0.02 | 0.60 | 0.03 | 0.03 | 0.390 |
| **0.6** | **0.1** | **0.4** | 1.50 | 0.02 | 0.02 | 2.10 | 0.02 | 0.02 | 0.60 | 0.03 | 0.03 | 0.390 |
| **0.6** | **0.2** | **0** | 1.50 | 0.02 | 0.02 | 2.10 | 0.02 | 0.02 | 0.60 | 0.03 | 0.03 | 0.372 |
| **0.6** | **0.2** | **0.2** | 1.50 | 0.02 | 0.02 | 2.10 | 0.02 | 0.02 | 0.60 | 0.03 | 0.03 | 0.999 |
| **0.6** | **0.2** | **0.4** | 1.50 | 0.02 | 0.02 | 2.10 | 0.02 | 0.02 | 0.60 | 0.03 | 0.03 | 0.390 |
|  |  |  | **Confounding model c** | | | | | | | | | |
|  |  |  | ${\hat{\boldsymbol{\beta}}}_{\boldsymbol{T\vert M=0}}$ | | | ${\hat{\boldsymbol{\beta}}}_{\boldsymbol{T\vert M=1}}$ | | | ${\hat{\boldsymbol{\beta}}}_{\boldsymbol{TM}}$ | | | |
| $\boldsymbol{\beta}_{\boldsymbol{TM}}$**^2^** | $\boldsymbol{\alpha}_{\boldsymbol{M}\boldsymbol{X}_{\boldsymbol{1}}}$**^3^** | $\boldsymbol{\beta}_{\boldsymbol{M}\boldsymbol{X}_{\boldsymbol{1}}}$**^4^** | **M** | $\mathbf{SE}_{\mathbf{E}}$ | $\mathbf{SE}_{\mathbf{M}}$ | **M** | $\mathbf{SE}_{\mathbf{E}}$ | $\mathbf{SE}_{\mathbf{M}}$ | **M** | $\mathbf{SE}_{\mathbf{E}}$ | $\mathbf{SE}_{\mathbf{M}}$ | **p^5^** |
| **0.3** | **0** | **0** | 1.50 | 0.02 | 0.02 | 1.80 | 0.02 | 0.02 | 0.30 | 0.03 | 0.03 | 0.390 |
| **0.3** | **0** | **0.2** | 1.50 | 0.02 | 0.02 | 1.80 | 0.02 | 0.02 | 0.30 | 0.03 | 0.03 | 0.408 |
| **0.3** | **0** | **0.4** | 1.50 | 0.02 | 0.02 | 1.80 | 0.02 | 0.02 | 0.30 | 0.03 | 0.03 | 0.999 |
| **0.3** | **0.1** | **0** | 1.50 | 0.02 | 0.02 | 1.80 | 0.02 | 0.02 | 0.30 | 0.03 | 0.03 | 0.408 |
| **0.3** | **0.1** | **0.2** | 1.50 | 0.02 | 0.02 | 1.80 | 0.02 | 0.02 | 0.30 | 0.03 | 0.03 | 0.999 |
| **0.3** | **0.1** | **0.4** | 1.50 | 0.02 | 0.02 | 1.80 | 0.02 | 0.02 | 0.30 | 0.03 | 0.03 | 0.425 |
| **0.3** | **0.2** | **0** | 1.50 | 0.02 | 0.02 | 1.80 | 0.02 | 0.02 | 0.30 | 0.03 | 0.03 | 0.999 |
| **0.3** | **0.2** | **0.2** | 1.50 | 0.02 | 0.02 | 1.80 | 0.02 | 0.02 | 0.30 | 0.03 | 0.03 | 0.111 |
| **0.3** | **0.2** | **0.4** | 1.50 | 0.02 | 0.02 | 1.80 | 0.02 | 0.02 | 0.30 | 0.03 | 0.03 | 0.999 |
| **0.6** | **0** | **0** | 1.50 | 0.02 | 0.02 | 2.10 | 0.02 | 0.02 | 0.60 | 0.03 | 0.03 | 0.098 |
| **0.6** | **0** | **0.2** | 1.50 | 0.02 | 0.02 | 2.10 | 0.02 | 0.02 | 0.60 | 0.03 | 0.03 | 0.999 |
| **0.6** | **0** | **0.4** | 1.50 | 0.02 | 0.02 | 2.10 | 0.02 | 0.02 | 0.60 | 0.03 | 0.03 | 0.372 |
| **0.6** | **0.1** | **0** | 1.50 | 0.02 | 0.02 | 2.10 | 0.02 | 0.02 | 0.60 | 0.03 | 0.03 | 0.999 |
| **0.6** | **0.1** | **0.2** | 1.50 | 0.02 | 0.02 | 2.10 | 0.02 | 0.02 | 0.60 | 0.03 | 0.03 | 0.408 |
| **0.6** | **0.1** | **0.4** | 1.50 | 0.02 | 0.02 | 2.10 | 0.02 | 0.02 | 0.60 | 0.03 | 0.03 | 0.408 |
| **0.6** | **0.2** | **0** | 1.50 | 0.02 | 0.02 | 2.10 | 0.02 | 0.02 | 0.60 | 0.03 | 0.03 | 0.372 |
| **0.6** | **0.2** | **0.2** | 1.50 | 0.02 | 0.02 | 2.10 | 0.02 | 0.02 | 0.60 | 0.03 | 0.03 | 0.999 |
| **0.6** | **0.2** | **0.4** | 1.50 | 0.02 | 0.02 | 2.10 | 0.02 | 0.02 | 0.60 | 0.03 | 0.03 | 0.390 |
|  |  |  | **Confounding model d** | | | | | | | | | |
|  |  |  | ${\hat{\boldsymbol{\beta}}}_{\boldsymbol{T\vert M=0}}$ | | | ${\hat{\boldsymbol{\beta}}}_{\boldsymbol{T\vert M=1}}$ | | | ${\hat{\boldsymbol{\beta}}}_{\boldsymbol{TM}}$ | | | |
| $\boldsymbol{\beta}_{\boldsymbol{TM}}$**^2^** | $\boldsymbol{\alpha}_{\boldsymbol{M}\boldsymbol{X}_{\boldsymbol{1}}}$**^3^** | $\boldsymbol{\beta}_{\boldsymbol{M}\boldsymbol{X}_{\boldsymbol{1}}}$**^4^** | **M** | $\mathbf{SE}_{\mathbf{E}}$ | $\mathbf{SE}_{\mathbf{M}}$ | **M** | $\mathbf{SE}_{\mathbf{E}}$ | $\mathbf{SE}_{\mathbf{M}}$ | **M** | $\mathbf{SE}_{\mathbf{E}}$ | $\mathbf{SE}_{\mathbf{M}}$ | **p^5^** |
| **0.3** | **0** | **0** | 1.50 | 0.02 | 0.02 | 1.80 | 0.02 | 0.02 | 0.30 | 0.03 | 0.03 | 0.390 |
| **0.3** | **0** | **0.2** | 1.50 | 0.02 | 0.02 | 1.80 | 0.02 | 0.02 | 0.30 | 0.03 | 0.03 | 0.408 |
| **0.3** | **0** | **0.4** | 1.50 | 0.02 | 0.02 | 1.80 | 0.02 | 0.02 | 0.30 | 0.03 | 0.03 | 0.999 |
| **0.3** | **0.1** | **0** | 1.50 | 0.02 | 0.02 | 1.80 | 0.02 | 0.02 | 0.30 | 0.03 | 0.03 | 0.408 |
| **0.3** | **0.1** | **0.2** | 1.50 | 0.02 | 0.02 | 1.80 | 0.02 | 0.02 | 0.30 | 0.03 | 0.03 | 0.999 |
| **0.3** | **0.1** | **0.4** | 1.50 | 0.02 | 0.02 | 1.80 | 0.02 | 0.02 | 0.30 | 0.03 | 0.03 | 0.425 |
| **0.3** | **0.2** | **0** | 1.50 | 0.02 | 0.02 | 1.80 | 0.02 | 0.02 | 0.30 | 0.03 | 0.03 | 0.999 |
| **0.3** | **0.2** | **0.2** | 1.50 | 0.02 | 0.02 | 1.80 | 0.02 | 0.02 | 0.30 | 0.03 | 0.03 | 0.111 |
| **0.3** | **0.2** | **0.4** | 1.50 | 0.02 | 0.02 | 1.80 | 0.02 | 0.02 | 0.30 | 0.03 | 0.03 | 0.999 |
| **0.6** | **0** | **0** | 1.50 | 0.02 | 0.02 | 2.10 | 0.02 | 0.02 | 0.60 | 0.03 | 0.03 | 0.098 |
| **0.6** | **0** | **0.2** | 1.50 | 0.02 | 0.02 | 2.10 | 0.02 | 0.02 | 0.60 | 0.03 | 0.03 | 0.999 |
| **0.6** | **0** | **0.4** | 1.50 | 0.02 | 0.02 | 2.10 | 0.02 | 0.02 | 0.60 | 0.03 | 0.03 | 0.372 |
| **0.6** | **0.1** | **0** | 1.50 | 0.02 | 0.02 | 2.10 | 0.02 | 0.02 | 0.60 | 0.03 | 0.03 | 0.999 |
| **0.6** | **0.1** | **0.2** | 1.50 | 0.02 | 0.02 | 2.10 | 0.02 | 0.02 | 0.60 | 0.03 | 0.03 | 0.408 |
| **0.6** | **0.1** | **0.4** | 1.50 | 0.02 | 0.02 | 2.10 | 0.02 | 0.02 | 0.60 | 0.03 | 0.03 | 0.408 |
| **0.6** | **0.2** | **0** | 1.50 | 0.02 | 0.02 | 2.10 | 0.02 | 0.02 | 0.60 | 0.03 | 0.03 | 0.372 |
| **0.6** | **0.2** | **0.2** | 1.50 | 0.02 | 0.02 | 2.10 | 0.02 | 0.02 | 0.60 | 0.03 | 0.03 | 0.999 |
| **0.6** | **0.2** | **0.4** | 1.50 | 0.02 | 0.02 | 2.10 | 0.02 | 0.02 | 0.60 | 0.03 | 0.03 | 0.390 |

^1^Confounding models: (A) adjusting for no moderator-confounders interactions, (B) adjusting for the one moderator-confounder interaction, (C) adjusting for all possible moderator-confounder interactions, (D) subgroup-specific confounding adjustment.
^2^$\beta_{TM}$: true magnitude of treatment effect modification
^3^$\alpha_{MX_{1}}$: magnitude of moderator-confounder interaction on treatment receipt
^4^$\beta_{MX_{1}}$: magnitude of moderator-confounder interaction on outcome
^5^p-value from a one-sample t-test comparing $\hat{\beta}_{TM}$ with the true value (0.3 or 0.6)

Table S2: Mean (M) and standard deviation (SD) of subgroup-specific treatment effects ($\hat{\beta}_{T|M=1}$ and $\hat{\beta}_{T|M=0}$) and treatment effect modification ($\hat{\beta}_{TM}$) estimated via propensity score covariate adjustment from the 500 simulated datasets where the moderator had a prevalence of 0.5

|  |  |  | **Confounding model a** | | | | | | | | | |
| --- | --- | --- | --- | --- | --- | --- | --- | --- | --- | --- | --- | --- |
|  |  |  | ${\hat{\boldsymbol{\beta}}}_{\boldsymbol{T\vert M=0}}$ | | | ${\hat{\boldsymbol{\beta}}}_{\boldsymbol{T\vert M=1}}$ | | | ${\hat{\boldsymbol{\beta}}}_{\boldsymbol{TM}}$ | | | |
| $\boldsymbol{\beta}_{\boldsymbol{TM}}$**^2^** | $\boldsymbol{\alpha}_{\boldsymbol{M}\boldsymbol{X}_{\boldsymbol{1}}}$**^3^** | $\boldsymbol{\beta}_{\boldsymbol{M}\boldsymbol{X}_{\boldsymbol{1}}}$**^4^** | **M** | $\mathbf{SE}_{\mathbf{E}}$ | $\mathbf{SE}_{\mathbf{M}}$ | **M** | $\mathbf{SE}_{\mathbf{E}}$ | $\mathbf{SE}_{\mathbf{M}}$ | **M** | $\mathbf{SE}_{\mathbf{E}}$ | $\mathbf{SE}_{\mathbf{M}}$ | **p^5^** |
| **0.3** | **0** | **0** | 1.49 | 0.04 | 0.06 | 1.81 | 0.04 | 0.06 | 0.31 | 0.08 | 0.08 | <0.001 |
| **0.3** | **0** | **0.2** | 1.46 | 0.04 | 0.06 | 1.84 | 0.05 | 0.06 | 0.38 | 0.08 | 0.08 | <0.001 |
| **0.3** | **0** | **0.4** | 1.43 | 0.04 | 0.06 | 1.87 | 0.05 | 0.06 | 0.44 | 0.08 | 0.09 | <0.001 |
| **0.3** | **0.1** | **0** | 1.49 | 0.04 | 0.06 | 1.81 | 0.04 | 0.06 | 0.31 | 0.08 | 0.08 | <0.001 |
| **0.3** | **0.1** | **0.2** | 1.46 | 0.04 | 0.06 | 1.85 | 0.05 | 0.06 | 0.38 | 0.09 | 0.08 | <0.001 |
| **0.3** | **0.1** | **0.4** | 1.43 | 0.04 | 0.06 | 1.89 | 0.05 | 0.06 | 0.46 | 0.08 | 0.08 | <0.001 |
| **0.3** | **0.2** | **0** | 1.50 | 0.04 | 0.06 | 1.80 | 0.04 | 0.06 | 0.3 | 0.08 | 0.08 | 0.276 |
| **0.3** | **0.2** | **0.2** | 1.47 | 0.04 | 0.06 | 1.85 | 0.05 | 0.06 | 0.38 | 0.08 | 0.08 | <0.001 |
| **0.3** | **0.2** | **0.4** | 1.44 | 0.05 | 0.06 | 1.91 | 0.05 | 0.06 | 0.47 | 0.09 | 0.08 | <0.001 |
| **0.6** | **0** | **0** | 1.49 | 0.04 | 0.06 | 2.11 | 0.04 | 0.06 | 0.61 | 0.08 | 0.08 | <0.001 |
| **0.6** | **0** | **0.2** | 1.46 | 0.04 | 0.06 | 2.14 | 0.05 | 0.06 | 0.68 | 0.08 | 0.08 | <0.001 |
| **0.6** | **0** | **0.4** | 1.44 | 0.04 | 0.06 | 2.17 | 0.05 | 0.06 | 0.73 | 0.09 | 0.09 | <0.001 |
| **0.6** | **0.1** | **0** | 1.49 | 0.04 | 0.06 | 2.11 | 0.05 | 0.06 | 0.62 | 0.08 | 0.08 | <0.001 |
| **0.6** | **0.1** | **0.2** | 1.47 | 0.04 | 0.06 | 2.15 | 0.04 | 0.06 | 0.68 | 0.08 | 0.08 | <0.001 |
| **0.6** | **0.1** | **0.4** | 1.44 | 0.04 | 0.06 | 2.19 | 0.05 | 0.06 | 0.76 | 0.08 | 0.08 | <0.001 |
| **0.6** | **0.2** | **0** | 1.50 | 0.04 | 0.06 | 2.10 | 0.05 | 0.06 | 0.60 | 0.08 | 0.08 | 0.999 |
| **0.6** | **0.2** | **0.2** | 1.47 | 0.05 | 0.06 | 2.15 | 0.05 | 0.06 | 0.68 | 0.09 | 0.08 | <0.001 |
| **0.6** | **0.2** | **0.4** | 1.44 | 0.04 | 0.06 | 2.21 | 0.05 | 0.06 | 0.77 | 0.08 | 0.08 | <0.001 |
|  |  |  | **Confounding model b** | | | | | | | | | |
|  |  |  | ${\hat{\boldsymbol{\beta}}}_{\boldsymbol{T\vert M=0}}$ | | | ${\hat{\boldsymbol{\beta}}}_{\boldsymbol{T\vert M=1}}$ | | | ${\hat{\boldsymbol{\beta}}}_{\boldsymbol{TM}}$ | | | |
| $\boldsymbol{\beta}_{\boldsymbol{TM}}$**^2^** | $\boldsymbol{\alpha}_{\boldsymbol{M}\boldsymbol{X}_{\boldsymbol{1}}}$**^3^** | $\boldsymbol{\beta}_{\boldsymbol{M}\boldsymbol{X}_{\boldsymbol{1}}}$**^4^** | **M** | $\mathbf{SE}_{\mathbf{E}}$ | $\mathbf{SE}_{\mathbf{M}}$ | **M** | $\mathbf{SE}_{\mathbf{E}}$ | $\mathbf{SE}_{\mathbf{M}}$ | **M** | $\mathbf{SE}_{\mathbf{E}}$ | $\mathbf{SE}_{\mathbf{M}}$ | **p^5^** |
| **0.3** | **0** | **0** | 1.49 | 0.05 | 0.06 | 1.81 | 0.05 | 0.06 | 0.31 | 0.10 | 0.08 | 0.004 |
| **0.3** | **0** | **0.2** | 1.46 | 0.05 | 0.06 | 1.84 | 0.05 | 0.06 | 0.38 | 0.10 | 0.08 | <0.001 |
| **0.3** | **0** | **0.4** | 1.44 | 0.05 | 0.06 | 1.87 | 0.05 | 0.06 | 0.43 | 0.10 | 0.09 | <0.001 |
| **0.3** | **0.1** | **0** | 1.52 | 0.05 | 0.06 | 1.78 | 0.06 | 0.06 | 0.27 | 0.11 | 0.08 | <0.001 |
| **0.3** | **0.1** | **0.2** | 1.48 | 0.05 | 0.06 | 1.82 | 0.06 | 0.06 | 0.33 | 0.11 | 0.08 | <0.001 |
| **0.3** | **0.1** | **0.4** | 1.45 | 0.05 | 0.06 | 1.85 | 0.06 | 0.06 | 0.40 | 0.11 | 0.08 | <0.001 |
| **0.3** | **0.2** | **0** | 1.54 | 0.06 | 0.06 | 1.76 | 0.06 | 0.06 | 0.22 | 0.11 | 0.08 | <0.001 |
| **0.3** | **0.2** | **0.2** | 1.51 | 0.06 | 0.06 | 1.79 | 0.06 | 0.06 | 0.29 | 0.11 | 0.08 | 0.003 |
| **0.3** | **0.2** | **0.4** | 1.47 | 0.06 | 0.06 | 1.83 | 0.06 | 0.06 | 0.36 | 0.12 | 0.08 | <0.001 |
| **0.6** | **0** | **0** | 1.49 | 0.05 | 0.06 | 2.11 | 0.05 | 0.06 | 0.61 | 0.10 | 0.08 | 0.001 |
| **0.6** | **0** | **0.2** | 1.46 | 0.05 | 0.06 | 2.14 | 0.05 | 0.06 | 0.68 | 0.09 | 0.08 | <0.001 |
| **0.6** | **0** | **0.4** | 1.44 | 0.05 | 0.06 | 2.16 | 0.05 | 0.06 | 0.72 | 0.10 | 0.09 | <0.001 |
| **0.6** | **0.1** | **0** | 1.51 | 0.05 | 0.06 | 2.09 | 0.06 | 0.06 | 0.58 | 0.11 | 0.08 | <0.001 |
| **0.6** | **0.1** | **0.2** | 1.48 | 0.06 | 0.06 | 2.12 | 0.06 | 0.06 | 0.63 | 0.11 | 0.08 | <0.001 |
| **0.6** | **0.1** | **0.4** | 1.45 | 0.05 | 0.06 | 2.15 | 0.06 | 0.06 | 0.70 | 0.10 | 0.08 | <0.001 |
| **0.6** | **0.2** | **0** | 1.54 | 0.05 | 0.06 | 2.06 | 0.06 | 0.06 | 0.52 | 0.11 | 0.08 | <0.001 |
| **0.6** | **0.2** | **0.2** | 1.51 | 0.06 | 0.06 | 2.09 | 0.06 | 0.06 | 0.58 | 0.11 | 0.08 | <0.001 |
| **0.6** | **0.2** | **0.4** | 1.47 | 0.06 | 0.06 | 2.13 | 0.06 | 0.06 | 0.66 | 0.11 | 0.08 | <0.001 |
|  |  |  | **Confounding model c** | | | | | | | | | |
|  |  |  | ${\hat{\boldsymbol{\beta}}}_{\boldsymbol{T\vert M=0}}$ | | | ${\hat{\boldsymbol{\beta}}}_{\boldsymbol{T\vert M=1}}$ | | | ${\hat{\boldsymbol{\beta}}}_{\boldsymbol{TM}}$ | | | |
| $\boldsymbol{\beta}_{\boldsymbol{TM}}$**^2^** | $\boldsymbol{\alpha}_{\boldsymbol{M}\boldsymbol{X}_{\boldsymbol{1}}}$**^3^** | $\boldsymbol{\beta}_{\boldsymbol{M}\boldsymbol{X}_{\boldsymbol{1}}}$**^4^** | **M** | $\mathbf{SE}_{\mathbf{E}}$ | $\mathbf{SE}_{\mathbf{M}}$ | **M** | $\mathbf{SE}_{\mathbf{E}}$ | $\mathbf{SE}_{\mathbf{M}}$ | **M** | $\mathbf{SE}_{\mathbf{E}}$ | $\mathbf{SE}_{\mathbf{M}}$ | **p^5^** |
| **0.3** | **0** | **0** | 1.50 | 0.06 | 0.06 | 1.81 | 0.06 | 0.06 | 0.31 | 0.11 | 0.09 | 0.024 |
| **0.3** | **0** | **0.2** | 1.47 | 0.06 | 0.06 | 1.84 | 0.06 | 0.06 | 0.37 | 0.12 | 0.09 | <0.001 |
| **0.3** | **0** | **0.4** | 1.44 | 0.06 | 0.07 | 1.87 | 0.06 | 0.07 | 0.43 | 0.12 | 0.09 | <0.001 |
| **0.3** | **0.1** | **0** | 1.51 | 0.05 | 0.06 | 1.78 | 0.06 | 0.06 | 0.27 | 0.11 | 0.09 | <0.001 |
| **0.3** | **0.1** | **0.2** | 1.48 | 0.06 | 0.06 | 1.82 | 0.06 | 0.06 | 0.34 | 0.12 | 0.09 | <0.001 |
| **0.3** | **0.1** | **0.4** | 1.45 | 0.06 | 0.06 | 1.85 | 0.07 | 0.07 | 0.40 | 0.12 | 0.09 | <0.001 |
| **0.3** | **0.2** | **0** | 1.54 | 0.06 | 0.06 | 1.76 | 0.06 | 0.07 | 0.23 | 0.11 | 0.09 | <0.001 |
| **0.3** | **0.2** | **0.2** | 1.50 | 0.06 | 0.06 | 1.80 | 0.07 | 0.06 | 0.29 | 0.12 | 0.09 | 0.268 |
| **0.3** | **0.2** | **0.4** | 1.47 | 0.06 | 0.06 | 1.84 | 0.07 | 0.06 | 0.37 | 0.13 | 0.09 | <0.001 |
| **0.6** | **0** | **0** | 1.50 | 0.06 | 0.06 | 2.10 | 0.06 | 0.06 | 0.61 | 0.11 | 0.09 | 0.101 |
| **0.6** | **0** | **0.2** | 1.46 | 0.05 | 0.06 | 2.14 | 0.06 | 0.06 | 0.68 | 0.11 | 0.09 | <0.001 |
| **0.6** | **0** | **0.4** | 1.44 | 0.06 | 0.07 | 2.16 | 0.06 | 0.07 | 0.72 | 0.12 | 0.09 | <0.001 |
| **0.6** | **0.1** | **0** | 1.51 | 0.06 | 0.06 | 2.09 | 0.06 | 0.06 | 0.58 | 0.11 | 0.09 | 0.001 |
| **0.6** | **0.1** | **0.2** | 1.48 | 0.06 | 0.06 | 2.12 | 0.06 | 0.06 | 0.64 | 0.12 | 0.09 | <0.001 |
| **0.6** | **0.1** | **0.4** | 1.45 | 0.06 | 0.06 | 2.15 | 0.06 | 0.06 | 0.70 | 0.12 | 0.09 | <0.001 |
| **0.6** | **0.2** | **0** | 1.53 | 0.05 | 0.06 | 2.06 | 0.06 | 0.07 | 0.53 | 0.11 | 0.09 | <0.001 |
| **0.6** | **0.2** | **0.2** | 1.51 | 0.06 | 0.06 | 2.09 | 0.06 | 0.06 | 0.59 | 0.12 | 0.09 | 0.041 |
| **0.6** | **0.2** | **0.4** | 1.47 | 0.06 | 0.06 | 2.14 | 0.06 | 0.06 | 0.67 | 0.12 | 0.09 | <0.001 |
|  |  |  | **Confounding model d** | | | | | | | | | |
|  |  |  | ${\hat{\boldsymbol{\beta}}}_{\boldsymbol{T\vert M=0}}$ | | | ${\hat{\boldsymbol{\beta}}}_{\boldsymbol{T\vert M=1}}$ | | | ${\hat{\boldsymbol{\beta}}}_{\boldsymbol{TM}}$ | | | |
| $\boldsymbol{\beta}_{\boldsymbol{TM}}$**^2^** | $\boldsymbol{\alpha}_{\boldsymbol{M}\boldsymbol{X}_{\boldsymbol{1}}}$**^3^** | $\boldsymbol{\beta}_{\boldsymbol{M}\boldsymbol{X}_{\boldsymbol{1}}}$**^4^** | **M** | $\mathbf{SE}_{\mathbf{E}}$ | $\mathbf{SE}_{\mathbf{M}}$ | **M** | $\mathbf{SE}_{\mathbf{E}}$ | $\mathbf{SE}_{\mathbf{M}}$ | **M** | $\mathbf{SE}_{\mathbf{E}}$ | $\mathbf{SE}_{\mathbf{M}}$ | **p^5^** |
| **0.3** | **0** | **0** | 1.50 | 0.02 | 0.06 | 1.80 | 0.02 | 0.06 | 0.30 | 0.03 | 0.09 | 0.425 |
| **0.3** | **0** | **0.2** | 1.50 | 0.02 | 0.06 | 1.80 | 0.02 | 0.07 | 0.30 | 0.03 | 0.09 | 0.425 |
| **0.3** | **0** | **0.4** | 1.50 | 0.02 | 0.06 | 1.80 | 0.02 | 0.07 | 0.30 | 0.03 | 0.09 | 0.999 |
| **0.3** | **0.1** | **0** | 1.50 | 0.02 | 0.06 | 1.80 | 0.02 | 0.06 | 0.30 | 0.03 | 0.09 | 0.441 |
| **0.3** | **0.1** | **0.2** | 1.50 | 0.02 | 0.06 | 1.80 | 0.02 | 0.06 | 0.30 | 0.03 | 0.09 | 0.999 |
| **0.3** | **0.1** | **0.4** | 1.50 | 0.02 | 0.06 | 1.80 | 0.02 | 0.07 | 0.30 | 0.03 | 0.09 | 0.441 |
| **0.3** | **0.2** | **0** | 1.50 | 0.02 | 0.06 | 1.80 | 0.02 | 0.07 | 0.30 | 0.03 | 0.09 | 0.999 |
| **0.3** | **0.2** | **0.2** | 1.50 | 0.02 | 0.06 | 1.80 | 0.02 | 0.06 | 0.30 | 0.03 | 0.09 | 0.124 |
| **0.3** | **0.2** | **0.4** | 1.50 | 0.02 | 0.06 | 1.80 | 0.02 | 0.07 | 0.30 | 0.03 | 0.09 | 0.999 |
| **0.6** | **0** | **0** | 1.50 | 0.02 | 0.06 | 2.10 | 0.02 | 0.06 | 0.60 | 0.03 | 0.09 | 0.111 |
| **0.6** | **0** | **0.2** | 1.50 | 0.02 | 0.06 | 2.10 | 0.02 | 0.07 | 0.60 | 0.03 | 0.09 | 0.441 |
| **0.6** | **0** | **0.4** | 1.50 | 0.02 | 0.06 | 2.10 | 0.02 | 0.07 | 0.60 | 0.03 | 0.09 | 0.098 |
| **0.6** | **0.1** | **0** | 1.50 | 0.02 | 0.06 | 2.10 | 0.02 | 0.06 | 0.60 | 0.03 | 0.09 | 0.999 |
| **0.6** | **0.1** | **0.2** | 1.50 | 0.02 | 0.06 | 2.10 | 0.02 | 0.06 | 0.60 | 0.03 | 0.09 | 0.999 |
| **0.6** | **0.1** | **0.4** | 1.50 | 0.02 | 0.06 | 2.10 | 0.02 | 0.07 | 0.60 | 0.03 | 0.09 | 0.441 |
| **0.6** | **0.2** | **0** | 1.50 | 0.02 | 0.06 | 2.10 | 0.02 | 0.07 | 0.60 | 0.03 | 0.09 | 0.39 |
| **0.6** | **0.2** | **0.2** | 1.50 | 0.02 | 0.06 | 2.10 | 0.02 | 0.07 | 0.60 | 0.03 | 0.09 | 0.441 |
| **0.6** | **0.2** | **0.4** | 1.50 | 0.02 | 0.06 | 2.10 | 0.02 | 0.07 | 0.60 | 0.03 | 0.09 | 0.425 |

^1^Confounding models: (A) adjusting for no moderator-confounders interactions, (B) adjusting for the one moderator-confounder interaction, (C) adjusting for all possible moderator-confounder interactions, (D) subgroup-specific confounding adjustment.
^2^$\beta_{TM}$: true magnitude of treatment effect modification
^3^$\alpha_{MX_{1}}$: magnitude of moderator-confounder interaction on treatment receipt
^4^$\beta_{MX_{1}}$: magnitude of moderator-confounder interaction on outcome
^5^p-value from a one-sample t-test comparing $\hat{\beta}_{TM}$ with the true value (0.3 or 0.6)

Table S3: Mean (M) and standard deviation (SD) of subgroup-specific treatment effects ($\hat{\beta}_{T|M=1}$ and $\hat{\beta}_{T|M=0}$) and treatment effect modification ($\hat{\beta}_{TM}$) estimated via inverse probability of treatment weighting from the 500 simulated datasets where the moderator had a prevalence of 0.5

|  |  |  | **Confounding model a** | | | | | | | | | |
| --- | --- | --- | --- | --- | --- | --- | --- | --- | --- | --- | --- | --- |
|  |  |  | ${\hat{\boldsymbol{\beta}}}_{\boldsymbol{T\vert M=0}}$ | | | ${\hat{\boldsymbol{\beta}}}_{\boldsymbol{T\vert M=1}}$ | | | ${\hat{\boldsymbol{\beta}}}_{\boldsymbol{TM}}$ | | | |
| $\boldsymbol{\beta}_{\boldsymbol{TM}}$**^2^** | $\boldsymbol{\alpha}_{\boldsymbol{M}\boldsymbol{X}_{\boldsymbol{1}}}$**^3^** | $\boldsymbol{\beta}_{\boldsymbol{M}\boldsymbol{X}_{\boldsymbol{1}}}$**^4^** | **M** | $\mathbf{SE}_{\mathbf{E}}$ | $\mathbf{SE}_{\mathbf{M}}$ | **M** | $\mathbf{SE}_{\mathbf{E}}$ | $\mathbf{SE}_{\mathbf{M}}$ | **M** | $\mathbf{SE}_{\mathbf{E}}$ | $\mathbf{SE}_{\mathbf{M}}$ | **p^5^** |
| **0.3** | **0** | **0** | 1.51 | 0.07 | 0.10 | 1.80 | 0.07 | 0.10 | 0.29 | 0.14 | 0.14 | 0.103 |
| **0.3** | **0** | **0.2** | 1.50 | 0.07 | 0.10 | 1.81 | 0.09 | 0.11 | 0.31 | 0.15 | 0.15 | 0.243 |
| **0.3** | **0** | **0.4** | 1.50 | 0.07 | 0.10 | 1.81 | 0.09 | 0.13 | 0.31 | 0.16 | 0.16 | 0.152 |
| **0.3** | **0.1** | **0** | 1.47 | 0.07 | 0.10 | 1.83 | 0.07 | 0.10 | 0.36 | 0.14 | 0.14 | <0.001 |
| **0.3** | **0.1** | **0.2** | 1.48 | 0.08 | 0.10 | 1.83 | 0.09 | 0.12 | 0.36 | 0.16 | 0.15 | <0.001 |
| **0.3** | **0.1** | **0.4** | 1.48 | 0.08 | 0.10 | 1.85 | 0.10 | 0.13 | 0.37 | 0.17 | 0.16 | <0.001 |
| **0.3** | **0.2** | **0** | 1.45 | 0.07 | 0.10 | 1.86 | 0.08 | 0.10 | 0.40 | 0.14 | 0.14 | <0.001 |
| **0.3** | **0.2** | **0.2** | 1.45 | 0.08 | 0.10 | 1.87 | 0.09 | 0.11 | 0.42 | 0.16 | 0.15 | <0.001 |
| **0.3** | **0.2** | **0.4** | 1.45 | 0.07 | 0.10 | 1.90 | 0.09 | 0.13 | 0.45 | 0.16 | 0.16 | <0.001 |
| **0.6** | **0** | **0** | 1.50 | 0.07 | 0.10 | 2.10 | 0.08 | 0.10 | 0.60 | 0.14 | 0.14 | 0.520 |
| **0.6** | **0** | **0.2** | 1.50 | 0.07 | 0.10 | 2.10 | 0.09 | 0.11 | 0.60 | 0.15 | 0.15 | 0.770 |
| **0.6** | **0** | **0.4** | 1.50 | 0.07 | 0.10 | 2.10 | 0.09 | 0.13 | 0.60 | 0.15 | 0.16 | 0.450 |
| **0.6** | **0.1** | **0** | 1.48 | 0.07 | 0.10 | 2.12 | 0.08 | 0.10 | 0.65 | 0.15 | 0.14 | <0.001 |
| **0.6** | **0.1** | **0.2** | 1.48 | 0.07 | 0.10 | 2.14 | 0.08 | 0.11 | 0.66 | 0.15 | 0.15 | <0.001 |
| **0.6** | **0.1** | **0.4** | 1.48 | 0.07 | 0.10 | 2.15 | 0.10 | 0.13 | 0.67 | 0.16 | 0.16 | <0.001 |
| **0.6** | **0.2** | **0** | 1.46 | 0.07 | 0.10 | 2.15 | 0.08 | 0.10 | 0.69 | 0.15 | 0.14 | <0.001 |
| **0.6** | **0.2** | **0.2** | 1.45 | 0.07 | 0.10 | 2.17 | 0.09 | 0.11 | 0.72 | 0.16 | 0.15 | <0.001 |
| **0.6** | **0.2** | **0.4** | 1.45 | 0.07 | 0.10 | 2.20 | 0.09 | 0.13 | 0.75 | 0.16 | 0.16 | <0.001 |
|  |  |  | **Confounding model b** | | | | | | | | | |
|  |  |  | ${\hat{\boldsymbol{\beta}}}_{\boldsymbol{T\vert M=0}}$ | | | ${\hat{\boldsymbol{\beta}}}_{\boldsymbol{T\vert M=1}}$ | | | ${\hat{\boldsymbol{\beta}}}_{\boldsymbol{TM}}$ | | | |
| $\boldsymbol{\beta}_{\boldsymbol{TM}}$**^2^** | $\boldsymbol{\alpha}_{\boldsymbol{M}\boldsymbol{X}_{\boldsymbol{1}}}$**^3^** | $\boldsymbol{\beta}_{\boldsymbol{M}\boldsymbol{X}_{\boldsymbol{1}}}$**^4^** | **M** | $\mathbf{SE}_{\mathbf{E}}$ | $\mathbf{SE}_{\mathbf{M}}$ | **M** | $\mathbf{SE}_{\mathbf{E}}$ | $\mathbf{SE}_{\mathbf{M}}$ | **M** | $\mathbf{SE}_{\mathbf{E}}$ | $\mathbf{SE}_{\mathbf{M}}$ | **p^5^** |
| **0.3** | **0** | **0** | 1.51 | 0.06 | 0.10 | 1.80 | 0.07 | 0.10 | 0.29 | 0.12 | 0.14 | 0.150 |
| **0.3** | **0** | **0.2** | 1.50 | 0.06 | 0.10 | 1.80 | 0.07 | 0.11 | 0.31 | 0.12 | 0.15 | 0.280 |
| **0.3** | **0** | **0.4** | 1.50 | 0.06 | 0.10 | 1.80 | 0.07 | 0.13 | 0.30 | 0.12 | 0.16 | 0.857 |
| **0.3** | **0.1** | **0** | 1.50 | 0.06 | 0.10 | 1.80 | 0.07 | 0.10 | 0.31 | 0.12 | 0.14 | 0.272 |
| **0.3** | **0.1** | **0.2** | 1.50 | 0.07 | 0.10 | 1.80 | 0.08 | 0.12 | 0.30 | 0.13 | 0.15 | 0.505 |
| **0.3** | **0.1** | **0.4** | 1.50 | 0.07 | 0.10 | 1.80 | 0.08 | 0.13 | 0.30 | 0.14 | 0.16 | 0.619 |
| **0.3** | **0.2** | **0** | 1.50 | 0.06 | 0.10 | 1.81 | 0.07 | 0.11 | 0.31 | 0.12 | 0.15 | 0.207 |
| **0.3** | **0.2** | **0.2** | 1.50 | 0.07 | 0.10 | 1.80 | 0.08 | 0.12 | 0.31 | 0.14 | 0.16 | 0.418 |
| **0.3** | **0.2** | **0.4** | 1.50 | 0.06 | 0.10 | 1.81 | 0.08 | 0.14 | 0.31 | 0.13 | 0.17 | 0.117 |
| **0.6** | **0** | **0** | 1.50 | 0.06 | 0.10 | 2.10 | 0.07 | 0.10 | 0.60 | 0.12 | 0.14 | 0.714 |
| **0.6** | **0** | **0.2** | 1.50 | 0.07 | 0.10 | 2.10 | 0.07 | 0.11 | 0.60 | 0.13 | 0.15 | 0.866 |
| **0.6** | **0** | **0.4** | 1.50 | 0.06 | 0.10 | 2.10 | 0.07 | 0.13 | 0.60 | 0.12 | 0.16 | 0.460 |
| **0.6** | **0.1** | **0** | 1.50 | 0.06 | 0.10 | 2.10 | 0.07 | 0.10 | 0.60 | 0.13 | 0.14 | 0.485 |
| **0.6** | **0.1** | **0.2** | 1.50 | 0.06 | 0.10 | 2.10 | 0.07 | 0.12 | 0.60 | 0.13 | 0.15 | 0.859 |
| **0.6** | **0.1** | **0.4** | 1.50 | 0.06 | 0.10 | 2.10 | 0.07 | 0.13 | 0.60 | 0.12 | 0.16 | 0.719 |
| **0.6** | **0.2** | **0** | 1.50 | 0.06 | 0.10 | 2.10 | 0.07 | 0.11 | 0.60 | 0.13 | 0.15 | 0.598 |
| **0.6** | **0.2** | **0.2** | 1.50 | 0.06 | 0.10 | 2.10 | 0.08 | 0.12 | 0.60 | 0.13 | 0.16 | 0.498 |
| **0.6** | **0.2** | **0.4** | 1.50 | 0.06 | 0.10 | 2.11 | 0.08 | 0.13 | 0.61 | 0.12 | 0.17 | 0.150 |
|  |  |  | **Confounding model c** | | | | | | | | | |
|  |  |  | ${\hat{\boldsymbol{\beta}}}_{\boldsymbol{T\vert M=0}}$ | | | ${\hat{\boldsymbol{\beta}}}_{\boldsymbol{T\vert M=1}}$ | | | ${\hat{\boldsymbol{\beta}}}_{\boldsymbol{TM}}$ | | | |
| $\boldsymbol{\beta}_{\boldsymbol{TM}}$**^2^** | $\boldsymbol{\alpha}_{\boldsymbol{M}\boldsymbol{X}_{\boldsymbol{1}}}$**^3^** | $\boldsymbol{\beta}_{\boldsymbol{M}\boldsymbol{X}_{\boldsymbol{1}}}$**^4^** | **M** | $\mathbf{SE}_{\mathbf{E}}$ | $\mathbf{SE}_{\mathbf{M}}$ | **M** | $\mathbf{SE}_{\mathbf{E}}$ | $\mathbf{SE}_{\mathbf{M}}$ | **M** | $\mathbf{SE}_{\mathbf{E}}$ | $\mathbf{SE}_{\mathbf{M}}$ | **p^5^** |
| **0.3** | **0** | **0** | 1.50 | 0.03 | 0.10 | 1.80 | 0.03 | 0.10 | 0.30 | 0.04 | 0.14 | 0.999 |
| **0.3** | **0** | **0.2** | 1.50 | 0.03 | 0.10 | 1.80 | 0.03 | 0.12 | 0.30 | 0.04 | 0.15 | 0.276 |
| **0.3** | **0** | **0.4** | 1.50 | 0.03 | 0.10 | 1.80 | 0.04 | 0.13 | 0.30 | 0.04 | 0.16 | 0.603 |
| **0.3** | **0.1** | **0** | 1.50 | 0.02 | 0.10 | 1.80 | 0.03 | 0.11 | 0.30 | 0.04 | 0.15 | 0.999 |
| **0.3** | **0.1** | **0.2** | 1.50 | 0.02 | 0.10 | 1.80 | 0.04 | 0.12 | 0.30 | 0.05 | 0.16 | 0.999 |
| **0.3** | **0.1** | **0.4** | 1.50 | 0.03 | 0.10 | 1.80 | 0.04 | 0.13 | 0.30 | 0.05 | 0.17 | 0.331 |
| **0.3** | **0.2** | **0** | 1.50 | 0.02 | 0.10 | 1.80 | 0.04 | 0.11 | 0.30 | 0.04 | 0.15 | 0.603 |
| **0.3** | **0.2** | **0.2** | 1.50 | 0.02 | 0.10 | 1.80 | 0.04 | 0.12 | 0.30 | 0.05 | 0.16 | 0.172 |
| **0.3** | **0.2** | **0.4** | 1.50 | 0.02 | 0.10 | 1.80 | 0.04 | 0.14 | 0.30 | 0.05 | 0.17 | 0.180 |
| **0.6** | **0** | **0** | 1.50 | 0.03 | 0.10 | 2.10 | 0.03 | 0.10 | 0.60 | 0.04 | 0.14 | 0.240 |
| **0.6** | **0** | **0.2** | 1.50 | 0.02 | 0.10 | 2.10 | 0.03 | 0.12 | 0.60 | 0.04 | 0.15 | 0.287 |
| **0.6** | **0** | **0.4** | 1.50 | 0.02 | 0.10 | 2.10 | 0.03 | 0.13 | 0.60 | 0.04 | 0.16 | 0.102 |
| **0.6** | **0.1** | **0** | 1.50 | 0.02 | 0.10 | 2.10 | 0.03 | 0.10 | 0.60 | 0.04 | 0.14 | 0.567 |
| **0.6** | **0.1** | **0.2** | 1.50 | 0.02 | 0.10 | 2.10 | 0.03 | 0.12 | 0.60 | 0.04 | 0.15 | 0.576 |
| **0.6** | **0.1** | **0.4** | 1.50 | 0.02 | 0.10 | 2.10 | 0.04 | 0.13 | 0.60 | 0.05 | 0.17 | 0.145 |
| **0.6** | **0.2** | **0** | 1.50 | 0.02 | 0.10 | 2.10 | 0.04 | 0.11 | 0.60 | 0.04 | 0.15 | 0.310 |
| **0.6** | **0.2** | **0.2** | 1.50 | 0.03 | 0.10 | 2.10 | 0.04 | 0.12 | 0.60 | 0.05 | 0.16 | 0.163 |
| **0.6** | **0.2** | **0.4** | 1.50 | 0.02 | 0.10 | 2.10 | 0.05 | 0.14 | 0.60 | 0.05 | 0.17 | 0.086 |
|  |  |  | **Confounding model d** | | | | | | | | | |
|  |  |  | ${\hat{\boldsymbol{\beta}}}_{\boldsymbol{T\vert M=0}}$ | | | ${\hat{\boldsymbol{\beta}}}_{\boldsymbol{T\vert M=1}}$ | | | ${\hat{\boldsymbol{\beta}}}_{\boldsymbol{TM}}$ | | | |
| $\boldsymbol{\beta}_{\boldsymbol{TM}}$**^2^** | $\boldsymbol{\alpha}_{\boldsymbol{M}\boldsymbol{X}_{\boldsymbol{1}}}$**^3^** | $\boldsymbol{\beta}_{\boldsymbol{M}\boldsymbol{X}_{\boldsymbol{1}}}$**^4^** | **M** | $\mathbf{SE}_{\mathbf{E}}$ | $\mathbf{SE}_{\mathbf{M}}$ | **M** | $\mathbf{SE}_{\mathbf{E}}$ | $\mathbf{SE}_{\mathbf{M}}$ | **M** | $\mathbf{SE}_{\mathbf{E}}$ | $\mathbf{SE}_{\mathbf{M}}$ | **p^5^** |
| **0.3** | **0** | **0** | 1.50 | 0.03 | 0.10 | 1.80 | 0.03 | 0.10 | 0.30 | 0.04 | 0.14 | 0.999 |
| **0.3** | **0** | **0.2** | 1.50 | 0.03 | 0.10 | 1.80 | 0.03 | 0.12 | 0.30 | 0.04 | 0.15 | 0.276 |
| **0.3** | **0** | **0.4** | 1.50 | 0.03 | 0.10 | 1.80 | 0.04 | 0.13 | 0.30 | 0.04 | 0.16 | 0.603 |
| **0.3** | **0.1** | **0** | 1.50 | 0.02 | 0.10 | 1.80 | 0.03 | 0.11 | 0.30 | 0.04 | 0.15 | 0.999 |
| **0.3** | **0.1** | **0.2** | 1.50 | 0.02 | 0.10 | 1.80 | 0.04 | 0.12 | 0.30 | 0.05 | 0.16 | 0.999 |
| **0.3** | **0.1** | **0.4** | 1.50 | 0.03 | 0.10 | 1.80 | 0.04 | 0.13 | 0.30 | 0.05 | 0.17 | 0.331 |
| **0.3** | **0.2** | **0** | 1.50 | 0.02 | 0.10 | 1.80 | 0.04 | 0.11 | 0.30 | 0.04 | 0.15 | 0.603 |
| **0.3** | **0.2** | **0.2** | 1.50 | 0.02 | 0.10 | 1.80 | 0.04 | 0.12 | 0.30 | 0.05 | 0.16 | 0.172 |
| **0.3** | **0.2** | **0.4** | 1.50 | 0.02 | 0.10 | 1.80 | 0.04 | 0.14 | 0.30 | 0.05 | 0.17 | 0.180 |
| **0.6** | **0** | **0** | 1.50 | 0.03 | 0.10 | 2.10 | 0.03 | 0.10 | 0.60 | 0.04 | 0.14 | 0.240 |
| **0.6** | **0** | **0.2** | 1.50 | 0.02 | 0.10 | 2.10 | 0.03 | 0.12 | 0.60 | 0.04 | 0.15 | 0.287 |
| **0.6** | **0** | **0.4** | 1.50 | 0.02 | 0.10 | 2.10 | 0.03 | 0.13 | 0.60 | 0.04 | 0.16 | 0.102 |
| **0.6** | **0.1** | **0** | 1.50 | 0.02 | 0.10 | 2.10 | 0.03 | 0.10 | 0.60 | 0.04 | 0.14 | 0.567 |
| **0.6** | **0.1** | **0.2** | 1.50 | 0.02 | 0.10 | 2.10 | 0.03 | 0.12 | 0.60 | 0.04 | 0.15 | 0.576 |
| **0.6** | **0.1** | **0.4** | 1.50 | 0.02 | 0.10 | 2.10 | 0.04 | 0.13 | 0.60 | 0.05 | 0.17 | 0.145 |
| **0.6** | **0.2** | **0** | 1.50 | 0.02 | 0.10 | 2.10 | 0.04 | 0.11 | 0.60 | 0.04 | 0.15 | 0.310 |
| **0.6** | **0.2** | **0.2** | 1.50 | 0.03 | 0.10 | 2.10 | 0.04 | 0.12 | 0.60 | 0.05 | 0.16 | 0.163 |
| **0.6** | **0.2** | **0.4** | 1.50 | 0.02 | 0.10 | 2.10 | 0.05 | 0.14 | 0.60 | 0.05 | 0.17 | 0.086 |

^1^Confounding models: (A) adjusting for no moderator-confounders interactions, (B) adjusting for the one moderator-confounder interaction, (C) adjusting for all possible moderator-confounder interactions, (D) subgroup-specific confounding adjustment.
^2^$\beta_{TM}$: true magnitude of treatment effect modification
^3^$\alpha_{MX_{1}}$: magnitude of moderator-confounder interaction on treatment receipt
^4^$\beta_{MX_{1}}$: magnitude of moderator-confounder interaction on outcome
^5^p-value from a one-sample t-test comparing $\hat{\beta}_{TM}$ with the true value (0.3 or 0.6)

Table S4: Mean (M) and standard deviation (SD) of subgroup-specific treatment effects ($\hat{\beta}_{T|M=1}$ and $\hat{\beta}_{T|M=0}$) and treatment effect modification ($\hat{\beta}_{TM}$) estimated via propensity score matching weighting from the 500 simulated datasets where the moderator had a prevalence of 0.5

|  |  |  | **Confounding model a** | | | | | | | | | |
| --- | --- | --- | --- | --- | --- | --- | --- | --- | --- | --- | --- | --- |
|  |  |  | ${\hat{\boldsymbol{\beta}}}_{\boldsymbol{T\vert M=0}}$ | | | ${\hat{\boldsymbol{\beta}}}_{\boldsymbol{T\vert M=1}}$ | | | ${\hat{\boldsymbol{\beta}}}_{\boldsymbol{TM}}$ | | | |
| $\boldsymbol{\beta}_{\boldsymbol{TM}}$**^2^** | $\boldsymbol{\alpha}_{\boldsymbol{M}\boldsymbol{X}_{\boldsymbol{1}}}$**^3^** | $\boldsymbol{\beta}_{\boldsymbol{M}\boldsymbol{X}_{\boldsymbol{1}}}$**^4^** | **M** | $\mathbf{SE}_{\mathbf{E}}$ | $\mathbf{SE}_{\mathbf{M}}$ | **M** | $\mathbf{SE}_{\mathbf{E}}$ | $\mathbf{SE}_{\mathbf{M}}$ | **M** | $\mathbf{SE}_{\mathbf{E}}$ | $\mathbf{SE}_{\mathbf{M}}$ | **p^5^** |
| **0.3** | **0** | **0** | 1.50 | 0.10 | 0.13 | 1.80 | 0.09 | 0.13 | 0.30 | 0.17 | 0.18 | 0.788 |
| **0.3** | **0** | **0.2** | 1.50 | 0.10 | 0.13 | 1.81 | 0.10 | 0.14 | 0.32 | 0.18 | 0.19 | 0.044 |
| **0.3** | **0** | **0.4** | 1.50 | 0.10 | 0.13 | 1.81 | 0.11 | 0.16 | 0.31 | 0.19 | 0.21 | 0.125 |
| **0.3** | **0.1** | **0** | 1.47 | 0.10 | 0.13 | 1.83 | 0.09 | 0.13 | 0.36 | 0.18 | 0.19 | <0.001 |
| **0.3** | **0.1** | **0.2** | 1.49 | 0.11 | 0.13 | 1.83 | 0.10 | 0.15 | 0.34 | 0.20 | 0.20 | <0.001 |
| **0.3** | **0.1** | **0.4** | 1.48 | 0.11 | 0.13 | 1.85 | 0.12 | 0.16 | 0.37 | 0.22 | 0.21 | <0.001 |
| **0.3** | **0.2** | **0** | 1.46 | 0.10 | 0.13 | 1.84 | 0.10 | 0.14 | 0.39 | 0.18 | 0.19 | <0.001 |
| **0.3** | **0.2** | **0.2** | 1.46 | 0.11 | 0.13 | 1.86 | 0.11 | 0.15 | 0.40 | 0.19 | 0.20 | <0.001 |
| **0.3** | **0.2** | **0.4** | 1.46 | 0.11 | 0.13 | 1.88 | 0.12 | 0.17 | 0.42 | 0.20 | 0.21 | <0.001 |
| **0.6** | **0** | **0** | 1.51 | 0.10 | 0.13 | 2.10 | 0.09 | 0.13 | 0.60 | 0.17 | 0.18 | 0.590 |
| **0.6** | **0** | **0.2** | 1.50 | 0.10 | 0.13 | 2.11 | 0.10 | 0.15 | 0.61 | 0.18 | 0.19 | 0.326 |
| **0.6** | **0** | **0.4** | 1.51 | 0.10 | 0.13 | 2.10 | 0.10 | 0.16 | 0.60 | 0.18 | 0.21 | 0.535 |
| **0.6** | **0.1** | **0** | 1.48 | 0.10 | 0.13 | 2.12 | 0.10 | 0.13 | 0.64 | 0.18 | 0.19 | <0.001 |
| **0.6** | **0.1** | **0.2** | 1.48 | 0.10 | 0.13 | 2.14 | 0.10 | 0.15 | 0.66 | 0.18 | 0.20 | <0.001 |
| **0.6** | **0.1** | **0.4** | 1.48 | 0.10 | 0.13 | 2.15 | 0.12 | 0.16 | 0.67 | 0.20 | 0.21 | <0.001 |
| **0.6** | **0.2** | **0** | 1.47 | 0.11 | 0.13 | 2.13 | 0.10 | 0.13 | 0.66 | 0.19 | 0.19 | <0.001 |
| **0.6** | **0.2** | **0.2** | 1.46 | 0.11 | 0.14 | 2.16 | 0.11 | 0.15 | 0.70 | 0.20 | 0.20 | <0.001 |
| **0.6** | **0.2** | **0.4** | 1.46 | 0.11 | 0.13 | 2.18 | 0.11 | 0.17 | 0.72 | 0.20 | 0.21 | <0.001 |
|  |  |  | **Confounding model b** | | | | | | | | | |
|  |  |  | ${\hat{\boldsymbol{\beta}}}_{\boldsymbol{T\vert M=0}}$ | | | ${\hat{\boldsymbol{\beta}}}_{\boldsymbol{T\vert M=1}}$ | | | ${\hat{\boldsymbol{\beta}}}_{\boldsymbol{TM}}$ | | | |
| $\boldsymbol{\beta}_{\boldsymbol{TM}}$**^2^** | $\boldsymbol{\alpha}_{\boldsymbol{M}\boldsymbol{X}_{\boldsymbol{1}}}$**^3^** | $\boldsymbol{\beta}_{\boldsymbol{M}\boldsymbol{X}_{\boldsymbol{1}}}$**^4^** | **M** | $\mathbf{SE}_{\mathbf{E}}$ | $\mathbf{SE}_{\mathbf{M}}$ | **M** | $\mathbf{SE}_{\mathbf{E}}$ | $\mathbf{SE}_{\mathbf{M}}$ | **M** | $\mathbf{SE}_{\mathbf{E}}$ | $\mathbf{SE}_{\mathbf{M}}$ | **p^5^** |
| **0.3** | **0** | **0** | 1.51 | 0.09 | 0.13 | 1.80 | 0.09 | 0.13 | 0.30 | 0.16 | 0.18 | 0.496 |
| **0.3** | **0** | **0.2** | 1.49 | 0.10 | 0.13 | 1.82 | 0.09 | 0.14 | 0.32 | 0.17 | 0.19 | 0.007 |
| **0.3** | **0** | **0.4** | 1.50 | 0.10 | 0.13 | 1.81 | 0.10 | 0.16 | 0.31 | 0.18 | 0.21 | 0.310 |
| **0.3** | **0.1** | **0** | 1.50 | 0.10 | 0.13 | 1.81 | 0.09 | 0.13 | 0.31 | 0.17 | 0.18 | 0.237 |
| **0.3** | **0.1** | **0.2** | 1.50 | 0.10 | 0.13 | 1.80 | 0.09 | 0.15 | 0.30 | 0.17 | 0.20 | 0.999 |
| **0.3** | **0.1** | **0.4** | 1.50 | 0.09 | 0.13 | 1.81 | 0.09 | 0.16 | 0.30 | 0.16 | 0.21 | 0.783 |
| **0.3** | **0.2** | **0** | 1.50 | 0.10 | 0.13 | 1.81 | 0.09 | 0.14 | 0.31 | 0.16 | 0.19 | 0.493 |
| **0.3** | **0.2** | **0.2** | 1.50 | 0.10 | 0.13 | 1.81 | 0.10 | 0.15 | 0.31 | 0.18 | 0.20 | 0.380 |
| **0.3** | **0.2** | **0.4** | 1.49 | 0.10 | 0.13 | 1.82 | 0.10 | 0.17 | 0.32 | 0.18 | 0.21 | 0.006 |
| **0.6** | **0** | **0** | 1.51 | 0.09 | 0.13 | 2.11 | 0.09 | 0.13 | 0.60 | 0.16 | 0.18 | 0.783 |
| **0.6** | **0** | **0.2** | 1.50 | 0.10 | 0.13 | 2.11 | 0.09 | 0.14 | 0.62 | 0.17 | 0.19 | 0.050 |
| **0.6** | **0** | **0.4** | 1.50 | 0.10 | 0.13 | 2.10 | 0.10 | 0.16 | 0.60 | 0.17 | 0.21 | 0.697 |
| **0.6** | **0.1** | **0** | 1.50 | 0.10 | 0.13 | 2.11 | 0.09 | 0.13 | 0.61 | 0.17 | 0.18 | 0.084 |
| **0.6** | **0.1** | **0.2** | 1.50 | 0.10 | 0.13 | 2.10 | 0.09 | 0.15 | 0.60 | 0.17 | 0.20 | 0.692 |
| **0.6** | **0.1** | **0.4** | 1.50 | 0.10 | 0.13 | 2.11 | 0.10 | 0.17 | 0.61 | 0.17 | 0.21 | 0.122 |
| **0.6** | **0.2** | **0** | 1.50 | 0.10 | 0.13 | 2.11 | 0.09 | 0.13 | 0.60 | 0.16 | 0.19 | 0.681 |
| **0.6** | **0.2** | **0.2** | 1.51 | 0.10 | 0.13 | 2.11 | 0.09 | 0.15 | 0.60 | 0.17 | 0.20 | 0.789 |
| **0.6** | **0.2** | **0.4** | 1.50 | 0.10 | 0.13 | 2.11 | 0.09 | 0.17 | 0.61 | 0.16 | 0.21 | 0.408 |
|  |  |  | **Confounding model c** | | | | | | | | | |
|  |  |  | ${\hat{\boldsymbol{\beta}}}_{\boldsymbol{T\vert M=0}}$ | | | ${\hat{\boldsymbol{\beta}}}_{\boldsymbol{T\vert M=1}}$ | | | ${\hat{\boldsymbol{\beta}}}_{\boldsymbol{TM}}$ | | | |
| $\boldsymbol{\beta}_{\boldsymbol{TM}}$**^2^** | $\boldsymbol{\alpha}_{\boldsymbol{M}\boldsymbol{X}_{\boldsymbol{1}}}$**^3^** | $\boldsymbol{\beta}_{\boldsymbol{M}\boldsymbol{X}_{\boldsymbol{1}}}$**^4^** | **M** | $\mathbf{SE}_{\mathbf{E}}$ | $\mathbf{SE}_{\mathbf{M}}$ | **M** | $\mathbf{SE}_{\mathbf{E}}$ | $\mathbf{SE}_{\mathbf{M}}$ | **M** | $\mathbf{SE}_{\mathbf{E}}$ | $\mathbf{SE}_{\mathbf{M}}$ | **p^5^** |
| **0.3** | **0** | **0** | 1.50 | 0.08 | 0.13 | 1.81 | 0.07 | 0.13 | 0.31 | 0.13 | 0.18 | 0.394 |
| **0.3** | **0** | **0.2** | 1.51 | 0.08 | 0.13 | 1.80 | 0.08 | 0.15 | 0.30 | 0.13 | 0.19 | 0.729 |
| **0.3** | **0** | **0.4** | 1.51 | 0.08 | 0.13 | 1.81 | 0.09 | 0.16 | 0.30 | 0.15 | 0.21 | 0.879 |
| **0.3** | **0.1** | **0** | 1.50 | 0.08 | 0.13 | 1.81 | 0.07 | 0.13 | 0.31 | 0.13 | 0.19 | 0.117 |
| **0.3** | **0.1** | **0.2** | 1.50 | 0.08 | 0.13 | 1.81 | 0.08 | 0.15 | 0.31 | 0.13 | 0.20 | 0.163 |
| **0.3** | **0.1** | **0.4** | 1.50 | 0.08 | 0.13 | 1.80 | 0.09 | 0.17 | 0.30 | 0.14 | 0.21 | 0.526 |
| **0.3** | **0.2** | **0** | 1.51 | 0.08 | 0.13 | 1.81 | 0.08 | 0.14 | 0.30 | 0.12 | 0.19 | 0.857 |
| **0.3** | **0.2** | **0.2** | 1.50 | 0.09 | 0.13 | 1.80 | 0.08 | 0.15 | 0.30 | 0.14 | 0.20 | 0.871 |
| **0.3** | **0.2** | **0.4** | 1.50 | 0.08 | 0.13 | 1.81 | 0.08 | 0.17 | 0.31 | 0.14 | 0.21 | 0.342 |
| **0.6** | **0** | **0** | 1.51 | 0.08 | 0.13 | 2.10 | 0.08 | 0.13 | 0.59 | 0.13 | 0.18 | 0.051 |
| **0.6** | **0** | **0.2** | 1.51 | 0.08 | 0.13 | 2.10 | 0.08 | 0.15 | 0.60 | 0.14 | 0.20 | 0.869 |
| **0.6** | **0** | **0.4** | 1.51 | 0.08 | 0.13 | 2.10 | 0.09 | 0.16 | 0.59 | 0.14 | 0.21 | 0.051 |
| **0.6** | **0.1** | **0** | 1.51 | 0.08 | 0.13 | 2.10 | 0.07 | 0.13 | 0.60 | 0.12 | 0.18 | 0.573 |
| **0.6** | **0.1** | **0.2** | 1.50 | 0.09 | 0.13 | 2.11 | 0.08 | 0.15 | 0.61 | 0.14 | 0.20 | 0.071 |
| **0.6** | **0.1** | **0.4** | 1.50 | 0.08 | 0.13 | 2.11 | 0.08 | 0.17 | 0.61 | 0.14 | 0.21 | 0.186 |
| **0.6** | **0.2** | **0** | 1.50 | 0.08 | 0.13 | 2.11 | 0.07 | 0.14 | 0.61 | 0.13 | 0.19 | 0.211 |
| **0.6** | **0.2** | **0.2** | 1.50 | 0.09 | 0.13 | 2.11 | 0.08 | 0.15 | 0.60 | 0.14 | 0.20 | 0.523 |
| **0.6** | **0.2** | **0.4** | 1.50 | 0.08 | 0.13 | 2.12 | 0.09 | 0.17 | 0.62 | 0.14 | 0.21 | 0.016 |
|  |  |  | **Confounding model d** | | | | | | | | | |
|  |  |  | ${\hat{\boldsymbol{\beta}}}_{\boldsymbol{T\vert M=0}}$ | | | ${\hat{\boldsymbol{\beta}}}_{\boldsymbol{T\vert M=1}}$ | | | ${\hat{\boldsymbol{\beta}}}_{\boldsymbol{TM}}$ | | | |
| $\boldsymbol{\beta}_{\boldsymbol{TM}}$**^2^** | $\boldsymbol{\alpha}_{\boldsymbol{M}\boldsymbol{X}_{\boldsymbol{1}}}$**^3^** | $\boldsymbol{\beta}_{\boldsymbol{M}\boldsymbol{X}_{\boldsymbol{1}}}$**^4^** | **M** | $\mathbf{SE}_{\mathbf{E}}$ | $\mathbf{SE}_{\mathbf{M}}$ | **M** | $\mathbf{SE}_{\mathbf{E}}$ | $\mathbf{SE}_{\mathbf{M}}$ | **M** | $\mathbf{SE}_{\mathbf{E}}$ | $\mathbf{SE}_{\mathbf{M}}$ | **p^5^** |
| **0.3** | **0** | **0** | 1.51 | 0.05 | 0.13 | 1.81 | 0.06 | 0.13 | 0.30 | 0.08 | 0.18 | 0.576 |
| **0.3** | **0** | **0.2** | 1.50 | 0.06 | 0.13 | 1.81 | 0.06 | 0.15 | 0.30 | 0.08 | 0.19 | 0.246 |
| **0.3** | **0** | **0.4** | 1.51 | 0.06 | 0.13 | 1.81 | 0.06 | 0.16 | 0.30 | 0.08 | 0.21 | 0.595 |
| **0.3** | **0.1** | **0** | 1.50 | 0.06 | 0.13 | 1.81 | 0.06 | 0.13 | 0.31 | 0.09 | 0.18 | 0.069 |
| **0.3** | **0.1** | **0.2** | 1.51 | 0.05 | 0.13 | 1.81 | 0.06 | 0.15 | 0.31 | 0.08 | 0.20 | 0.163 |
| **0.3** | **0.1** | **0.4** | 1.50 | 0.05 | 0.13 | 1.81 | 0.06 | 0.17 | 0.30 | 0.08 | 0.21 | 0.287 |
| **0.3** | **0.2** | **0** | 1.51 | 0.06 | 0.13 | 1.81 | 0.07 | 0.14 | 0.31 | 0.09 | 0.19 | 0.205 |
| **0.3** | **0.2** | **0.2** | 1.51 | 0.06 | 0.13 | 1.81 | 0.06 | 0.15 | 0.30 | 0.09 | 0.20 | 0.795 |
| **0.3** | **0.2** | **0.4** | 1.50 | 0.05 | 0.13 | 1.81 | 0.06 | 0.17 | 0.31 | 0.08 | 0.21 | 0.030 |
| **0.6** | **0** | **0** | 1.51 | 0.05 | 0.13 | 2.11 | 0.06 | 0.13 | 0.60 | 0.08 | 0.18 | 0.168 |
| **0.6** | **0** | **0.2** | 1.50 | 0.05 | 0.13 | 2.11 | 0.06 | 0.15 | 0.61 | 0.08 | 0.19 | 0.158 |
| **0.6** | **0** | **0.4** | 1.50 | 0.05 | 0.13 | 2.10 | 0.07 | 0.16 | 0.60 | 0.08 | 0.21 | 0.788 |
| **0.6** | **0.1** | **0** | 1.51 | 0.06 | 0.13 | 2.11 | 0.06 | 0.13 | 0.60 | 0.08 | 0.18 | 0.287 |
| **0.6** | **0.1** | **0.2** | 1.50 | 0.06 | 0.13 | 2.11 | 0.06 | 0.15 | 0.61 | 0.08 | 0.20 | 0.003 |
| **0.6** | **0.1** | **0.4** | 1.51 | 0.05 | 0.13 | 2.12 | 0.06 | 0.16 | 0.61 | 0.09 | 0.21 | 0.073 |
| **0.6** | **0.2** | **0** | 1.51 | 0.06 | 0.13 | 2.11 | 0.06 | 0.14 | 0.60 | 0.08 | 0.19 | 0.790 |
| **0.6** | **0.2** | **0.2** | 1.51 | 0.06 | 0.13 | 2.11 | 0.06 | 0.15 | 0.60 | 0.09 | 0.20 | 0.304 |
| **0.6** | **0.2** | **0.4** | 1.50 | 0.06 | 0.13 | 2.12 | 0.06 | 0.17 | 0.61 | 0.08 | 0.21 | 0.001 |

^1^Confounding models: (A) adjusting for no moderator-confounders interactions, (B) adjusting for the one moderator-confounder interaction, (C) adjusting for all possible moderator-confounder interactions, (D) subgroup-specific confounding adjustment.
^2^$\beta_{TM}$: true magnitude of treatment effect modification
^3^$\alpha_{MX_{1}}$: magnitude of moderator-confounder interaction on treatment receipt
^4^$\beta_{MX_{1}}$: magnitude of moderator-confounder interaction on outcome
^5^p-value from a one-sample t-test comparing $\hat{\beta}_{TM}$ with the true value (0.3 or 0.6)

Table S5: Mean (M) and standard deviation (SD) of subgroup-specific treatment effects ($\hat{\beta}_{T|M=1}$ and $\hat{\beta}_{T|M=0}$) and treatment effect modification ($\hat{\beta}_{TM}$) estimated via regression adjustment from the 500 simulated datasets where the moderator had a prevalence of 0.1

|  |  |  | **Confounding model a** | | | | | | | | | |
| --- | --- | --- | --- | --- | --- | --- | --- | --- | --- | --- | --- | --- |
|  |  |  | ${\hat{\boldsymbol{\beta}}}_{\boldsymbol{T\vert M=0}}$ | | | ${\hat{\boldsymbol{\beta}}}_{\boldsymbol{T\vert M=1}}$ | | | ${\hat{\boldsymbol{\beta}}}_{\boldsymbol{TM}}$ | | | |
| $\boldsymbol{\beta}_{\boldsymbol{TM}}$**^2^** | $\boldsymbol{\alpha}_{\boldsymbol{M}\boldsymbol{X}_{\boldsymbol{1}}}$**^3^** | $\boldsymbol{\beta}_{\boldsymbol{M}\boldsymbol{X}_{\boldsymbol{1}}}$**^4^** | **M** | $\mathbf{SE}_{\mathbf{E}}$ | $\mathbf{SE}_{\mathbf{M}}$ | **M** | $\mathbf{SE}_{\mathbf{E}}$ | $\mathbf{SE}_{\mathbf{M}}$ | **M** | $\mathbf{SE}_{\mathbf{E}}$ | $\mathbf{SE}_{\mathbf{M}}$ | **p^5^** |
| **0.3** | **0** | **0** | 1.50 | 0.01 | 0.01 | 1.80 | 0.04 | 0.04 | 0.30 | 0.04 | 0.04 | 0.128 |
| **0.3** | **0** | **0.2** | 1.49 | 0.01 | 0.01 | 1.85 | 0.05 | 0.04 | 0.36 | 0.06 | 0.05 | <0.001 |
| **0.3** | **0** | **0.4** | 1.49 | 0.01 | 0.02 | 1.91 | 0.09 | 0.05 | 0.42 | 0.09 | 0.05 | <0.001 |
| **0.3** | **0.1** | **0** | 1.50 | 0.01 | 0.01 | 1.80 | 0.04 | 0.04 | 0.30 | 0.04 | 0.04 | 0.111 |
| **0.3** | **0.1** | **0.2** | 1.49 | 0.01 | 0.01 | 1.87 | 0.06 | 0.04 | 0.37 | 0.06 | 0.05 | <0.001 |
| **0.3** | **0.1** | **0.4** | 1.49 | 0.01 | 0.02 | 1.93 | 0.08 | 0.05 | 0.45 | 0.09 | 0.05 | <0.001 |
| **0.3** | **0.2** | **0** | 1.50 | 0.01 | 0.01 | 1.80 | 0.04 | 0.04 | 0.30 | 0.04 | 0.04 | 0.287 |
| **0.3** | **0.2** | **0.2** | 1.49 | 0.01 | 0.01 | 1.89 | 0.06 | 0.04 | 0.40 | 0.06 | 0.05 | <0.001 |
| **0.3** | **0.2** | **0.4** | 1.49 | 0.01 | 0.02 | 1.97 | 0.08 | 0.05 | 0.48 | 0.08 | 0.05 | <0.001 |
| **0.6** | **0** | **0** | 1.50 | 0.02 | 0.01 | 2.10 | 0.04 | 0.04 | 0.60 | 0.05 | 0.04 | 0.619 |
| **0.6** | **0** | **0.2** | 1.49 | 0.01 | 0.01 | 2.15 | 0.06 | 0.04 | 0.66 | 0.06 | 0.05 | <0.001 |
| **0.6** | **0** | **0.4** | 1.49 | 0.02 | 0.02 | 2.20 | 0.09 | 0.05 | 0.72 | 0.09 | 0.05 | <0.001 |
| **0.6** | **0.1** | **0** | 1.50 | 0.01 | 0.01 | 2.10 | 0.04 | 0.04 | 0.60 | 0.05 | 0.04 | 0.619 |
| **0.6** | **0.1** | **0.2** | 1.49 | 0.01 | 0.01 | 2.17 | 0.06 | 0.04 | 0.67 | 0.06 | 0.05 | <0.001 |
| **0.6** | **0.1** | **0.4** | 1.49 | 0.01 | 0.02 | 2.24 | 0.09 | 0.05 | 0.75 | 0.09 | 0.05 | <0.001 |
| **0.6** | **0.2** | **0** | 1.50 | 0.01 | 0.01 | 2.10 | 0.04 | 0.04 | 0.60 | 0.05 | 0.04 | 0.321 |
| **0.6** | **0.2** | **0.2** | 1.50 | 0.01 | 0.01 | 2.18 | 0.06 | 0.04 | 0.69 | 0.06 | 0.05 | <0.001 |
| **0.6** | **0.2** | **0.4** | 1.49 | 0.02 | 0.02 | 2.27 | 0.09 | 0.05 | 0.78 | 0.09 | 0.05 | <0.001 |
|  |  |  | **Confounding model b** | | | | | | | | | |
|  |  |  | ${\hat{\boldsymbol{\beta}}}_{\boldsymbol{T\vert M=0}}$ | | | ${\hat{\boldsymbol{\beta}}}_{\boldsymbol{T\vert M=1}}$ | | | ${\hat{\boldsymbol{\beta}}}_{\boldsymbol{TM}}$ | | | |
| $\boldsymbol{\beta}_{\boldsymbol{TM}}$**^2^** | $\boldsymbol{\alpha}_{\boldsymbol{M}\boldsymbol{X}_{\boldsymbol{1}}}$**^3^** | $\boldsymbol{\beta}_{\boldsymbol{M}\boldsymbol{X}_{\boldsymbol{1}}}$**^4^** | **M** | $\mathbf{SE}_{\mathbf{E}}$ | $\mathbf{SE}_{\mathbf{M}}$ | **M** | $\mathbf{SE}_{\mathbf{E}}$ | $\mathbf{SE}_{\mathbf{M}}$ | **M** | $\mathbf{SE}_{\mathbf{E}}$ | $\mathbf{SE}_{\mathbf{M}}$ | **p^5^** |
| **0.3** | **0** | **0** | 1.50 | 0.01 | 0.01 | 1.80 | 0.04 | 0.04 | 0.30 | 0.05 | 0.05 | 0.321 |
| **0.3** | **0** | **0.2** | 1.50 | 0.01 | 0.01 | 1.80 | 0.04 | 0.04 | 0.30 | 0.05 | 0.05 | 0.999 |
| **0.3** | **0** | **0.4** | 1.50 | 0.01 | 0.01 | 1.80 | 0.05 | 0.04 | 0.30 | 0.05 | 0.05 | 0.331 |
| **0.3** | **0.1** | **0** | 1.50 | 0.01 | 0.01 | 1.80 | 0.04 | 0.04 | 0.30 | 0.04 | 0.05 | 0.299 |
| **0.3** | **0.1** | **0.2** | 1.50 | 0.01 | 0.01 | 1.80 | 0.04 | 0.04 | 0.30 | 0.04 | 0.05 | 0.612 |
| **0.3** | **0.1** | **0.4** | 1.50 | 0.01 | 0.01 | 1.80 | 0.04 | 0.04 | 0.30 | 0.04 | 0.05 | 0.999 |
| **0.3** | **0.2** | **0** | 1.50 | 0.01 | 0.01 | 1.80 | 0.04 | 0.04 | 0.30 | 0.04 | 0.05 | 0.299 |
| **0.3** | **0.2** | **0.2** | 1.50 | 0.01 | 0.01 | 1.81 | 0.04 | 0.04 | 0.31 | 0.04 | 0.05 | 0.002 |
| **0.3** | **0.2** | **0.4** | 1.50 | 0.01 | 0.01 | 1.80 | 0.04 | 0.04 | 0.30 | 0.05 | 0.05 | 0.999 |
| **0.6** | **0** | **0** | 1.50 | 0.02 | 0.01 | 2.10 | 0.04 | 0.04 | 0.60 | 0.05 | 0.05 | 0.627 |
| **0.6** | **0** | **0.2** | 1.50 | 0.01 | 0.01 | 2.10 | 0.04 | 0.04 | 0.60 | 0.05 | 0.05 | 0.627 |
| **0.6** | **0** | **0.4** | 1.50 | 0.01 | 0.01 | 2.10 | 0.04 | 0.04 | 0.60 | 0.05 | 0.05 | 0.999 |
| **0.6** | **0.1** | **0** | 1.50 | 0.01 | 0.01 | 2.10 | 0.04 | 0.04 | 0.60 | 0.05 | 0.05 | 0.999 |
| **0.6** | **0.1** | **0.2** | 1.50 | 0.01 | 0.01 | 2.10 | 0.05 | 0.04 | 0.60 | 0.05 | 0.05 | 0.634 |
| **0.6** | **0.1** | **0.4** | 1.50 | 0.01 | 0.01 | 2.10 | 0.04 | 0.04 | 0.60 | 0.05 | 0.05 | 0.627 |
| **0.6** | **0.2** | **0** | 1.50 | 0.01 | 0.01 | 2.10 | 0.04 | 0.04 | 0.60 | 0.05 | 0.05 | 0.331 |
| **0.6** | **0.2** | **0.2** | 1.50 | 0.01 | 0.01 | 2.10 | 0.04 | 0.04 | 0.60 | 0.05 | 0.05 | 0.627 |
| **0.6** | **0.2** | **0.4** | 1.50 | 0.01 | 0.01 | 2.10 | 0.04 | 0.04 | 0.60 | 0.05 | 0.05 | 0.627 |
|  |  |  | **Confounding model c** | | | | | | | | | |
|  |  |  | ${\hat{\boldsymbol{\beta}}}_{\boldsymbol{T\vert M=0}}$ | | | ${\hat{\boldsymbol{\beta}}}_{\boldsymbol{T\vert M=1}}$ | | | ${\hat{\boldsymbol{\beta}}}_{\boldsymbol{TM}}$ | | | |
| $\boldsymbol{\beta}_{\boldsymbol{TM}}$**^2^** | $\boldsymbol{\alpha}_{\boldsymbol{M}\boldsymbol{X}_{\boldsymbol{1}}}$**^3^** | $\boldsymbol{\beta}_{\boldsymbol{M}\boldsymbol{X}_{\boldsymbol{1}}}$**^4^** | **M** | $\mathbf{SE}_{\mathbf{E}}$ | $\mathbf{SE}_{\mathbf{M}}$ | **M** | $\mathbf{SE}_{\mathbf{E}}$ | $\mathbf{SE}_{\mathbf{M}}$ | **M** | $\mathbf{SE}_{\mathbf{E}}$ | $\mathbf{SE}_{\mathbf{M}}$ | **p^5^** |
| **0.3** | **0** | **0** | 1.50 | 0.01 | 0.01 | 1.80 | 0.05 | 0.04 | 0.30 | 0.05 | 0.05 | 0.342 |
| **0.3** | **0** | **0.2** | 1.50 | 0.01 | 0.01 | 1.80 | 0.04 | 0.05 | 0.30 | 0.05 | 0.05 | 0.627 |
| **0.3** | **0** | **0.4** | 1.50 | 0.01 | 0.01 | 1.80 | 0.05 | 0.04 | 0.30 | 0.05 | 0.05 | 0.642 |
| **0.3** | **0.1** | **0** | 1.50 | 0.01 | 0.01 | 1.80 | 0.04 | 0.05 | 0.30 | 0.04 | 0.05 | 0.310 |
| **0.3** | **0.1** | **0.2** | 1.50 | 0.01 | 0.01 | 1.80 | 0.04 | 0.04 | 0.30 | 0.05 | 0.05 | 0.627 |
| **0.3** | **0.1** | **0.4** | 1.50 | 0.01 | 0.01 | 1.80 | 0.04 | 0.04 | 0.30 | 0.05 | 0.05 | 0.619 |
| **0.3** | **0.2** | **0** | 1.50 | 0.01 | 0.01 | 1.80 | 0.04 | 0.05 | 0.30 | 0.05 | 0.05 | 0.619 |
| **0.3** | **0.2** | **0.2** | 1.50 | 0.01 | 0.01 | 1.81 | 0.04 | 0.05 | 0.31 | 0.05 | 0.05 | 0.001 |
| **0.3** | **0.2** | **0.4** | 1.50 | 0.01 | 0.01 | 1.80 | 0.05 | 0.05 | 0.30 | 0.05 | 0.05 | 0.999 |
| **0.6** | **0** | **0** | 1.50 | 0.02 | 0.01 | 2.10 | 0.05 | 0.04 | 0.60 | 0.05 | 0.05 | 0.999 |
| **0.6** | **0** | **0.2** | 1.50 | 0.01 | 0.01 | 2.10 | 0.05 | 0.05 | 0.60 | 0.05 | 0.05 | 0.999 |
| **0.6** | **0** | **0.4** | 1.50 | 0.01 | 0.01 | 2.10 | 0.05 | 0.04 | 0.60 | 0.05 | 0.05 | 0.634 |
| **0.6** | **0.1** | **0** | 1.50 | 0.01 | 0.01 | 2.10 | 0.04 | 0.05 | 0.60 | 0.05 | 0.05 | 0.999 |
| **0.6** | **0.1** | **0.2** | 1.50 | 0.01 | 0.01 | 2.10 | 0.05 | 0.05 | 0.60 | 0.05 | 0.05 | 0.642 |
| **0.6** | **0.1** | **0.4** | 1.50 | 0.01 | 0.01 | 2.10 | 0.05 | 0.05 | 0.60 | 0.05 | 0.05 | 0.634 |
| **0.6** | **0.2** | **0** | 1.50 | 0.01 | 0.01 | 2.10 | 0.05 | 0.05 | 0.60 | 0.05 | 0.05 | 0.154 |
| **0.6** | **0.2** | **0.2** | 1.50 | 0.01 | 0.01 | 2.10 | 0.05 | 0.05 | 0.60 | 0.05 | 0.05 | 0.634 |
| **0.6** | **0.2** | **0.4** | 1.50 | 0.01 | 0.01 | 2.10 | 0.04 | 0.05 | 0.60 | 0.05 | 0.05 | 0.634 |
|  |  |  | **Confounding model d** | | | | | | | | | |
|  |  |  | ${\hat{\boldsymbol{\beta}}}_{\boldsymbol{T\vert M=0}}$ | | | ${\hat{\boldsymbol{\beta}}}_{\boldsymbol{T\vert M=1}}$ | | | ${\hat{\boldsymbol{\beta}}}_{\boldsymbol{TM}}$ | | | |
| $\boldsymbol{\beta}_{\boldsymbol{TM}}$**^2^** | $\boldsymbol{\alpha}_{\boldsymbol{M}\boldsymbol{X}_{\boldsymbol{1}}}$**^3^** | $\boldsymbol{\beta}_{\boldsymbol{M}\boldsymbol{X}_{\boldsymbol{1}}}$**^4^** | **M** | $\mathbf{SE}_{\mathbf{E}}$ | $\mathbf{SE}_{\mathbf{M}}$ | **M** | $\mathbf{SE}_{\mathbf{E}}$ | $\mathbf{SE}_{\mathbf{M}}$ | **M** | $\mathbf{SE}_{\mathbf{E}}$ | $\mathbf{SE}_{\mathbf{M}}$ | **p^5^** |
| **0.3** | **0** | **0** | 1.50 | 0.01 | 0.01 | 1.80 | 0.05 | 0.04 | 0.30 | 0.05 | 0.05 | 0.342 |
| **0.3** | **0** | **0.2** | 1.50 | 0.01 | 0.01 | 1.80 | 0.04 | 0.04 | 0.30 | 0.05 | 0.05 | 0.627 |
| **0.3** | **0** | **0.4** | 1.50 | 0.01 | 0.01 | 1.80 | 0.05 | 0.04 | 0.30 | 0.05 | 0.05 | 0.642 |
| **0.3** | **0.1** | **0** | 1.50 | 0.01 | 0.01 | 1.80 | 0.04 | 0.05 | 0.30 | 0.04 | 0.05 | 0.310 |
| **0.3** | **0.1** | **0.2** | 1.50 | 0.01 | 0.01 | 1.80 | 0.04 | 0.04 | 0.30 | 0.05 | 0.05 | 0.627 |
| **0.3** | **0.1** | **0.4** | 1.50 | 0.01 | 0.01 | 1.80 | 0.04 | 0.04 | 0.30 | 0.05 | 0.05 | 0.619 |
| **0.3** | **0.2** | **0** | 1.50 | 0.01 | 0.01 | 1.80 | 0.04 | 0.05 | 0.30 | 0.05 | 0.05 | 0.619 |
| **0.3** | **0.2** | **0.2** | 1.50 | 0.01 | 0.01 | 1.81 | 0.04 | 0.05 | 0.31 | 0.05 | 0.05 | 0.001 |
| **0.3** | **0.2** | **0.4** | 1.50 | 0.01 | 0.01 | 1.80 | 0.05 | 0.05 | 0.30 | 0.05 | 0.05 | 0.999 |
| **0.6** | **0** | **0** | 1.50 | 0.02 | 0.01 | 2.10 | 0.05 | 0.04 | 0.60 | 0.05 | 0.05 | 0.999 |
| **0.6** | **0** | **0.2** | 1.50 | 0.01 | 0.01 | 2.10 | 0.05 | 0.05 | 0.60 | 0.05 | 0.05 | 0.999 |
| **0.6** | **0** | **0.4** | 1.50 | 0.01 | 0.01 | 2.10 | 0.05 | 0.04 | 0.60 | 0.05 | 0.05 | 0.634 |
| **0.6** | **0.1** | **0** | 1.50 | 0.01 | 0.01 | 2.10 | 0.04 | 0.05 | 0.60 | 0.05 | 0.05 | 0.999 |
| **0.6** | **0.1** | **0.2** | 1.50 | 0.01 | 0.01 | 2.10 | 0.05 | 0.05 | 0.60 | 0.05 | 0.05 | 0.642 |
| **0.6** | **0.1** | **0.4** | 1.50 | 0.01 | 0.01 | 2.10 | 0.05 | 0.05 | 0.60 | 0.05 | 0.05 | 0.634 |
| **0.6** | **0.2** | **0** | 1.50 | 0.01 | 0.01 | 2.10 | 0.05 | 0.05 | 0.60 | 0.05 | 0.05 | 0.154 |
| **0.6** | **0.2** | **0.2** | 1.50 | 0.01 | 0.01 | 2.10 | 0.05 | 0.05 | 0.60 | 0.05 | 0.05 | 0.634 |
| **0.6** | **0.2** | **0.4** | 1.50 | 0.01 | 0.01 | 2.10 | 0.04 | 0.05 | 0.60 | 0.05 | 0.05 | 0.634 |

^1^Confounding models: (A) adjusting for no moderator-confounders interactions, (B) adjusting for the one moderator-confounder interaction, (C) adjusting for all possible moderator-confounder interactions, (D) subgroup-specific confounding adjustment.
^2^$\beta_{TM}$: true magnitude of treatment effect modification
^3^$\alpha_{MX_{1}}$: magnitude of moderator-confounder interaction on treatment receipt
^4^$\beta_{MX_{1}}$: magnitude of moderator-confounder interaction on outcome
^5^p-value from a one-sample t-test comparing $\hat{\beta}_{TM}$ with the true value (0.3 or 0.6)

Table S6: Mean (M) and standard deviation (SD) of subgroup-specific treatment effects ($\hat{\beta}_{T|M=1}$ and $\hat{\beta}_{T|M=0}$) and treatment effect modification ($\hat{\beta}_{TM}$) estimated via propensity score covariate adjustment from the 500 simulated datasets where the moderator had a prevalence of 0.1

|  |  |  | **Confounding model a** | | | | | | | | | |
| --- | --- | --- | --- | --- | --- | --- | --- | --- | --- | --- | --- | --- |
|  |  |  | ${\hat{\boldsymbol{\beta}}}_{\boldsymbol{T\vert M=0}}$ | | | ${\hat{\boldsymbol{\beta}}}_{\boldsymbol{T\vert M=1}}$ | | | ${\hat{\boldsymbol{\beta}}}_{\boldsymbol{TM}}$ | | | |
| $\boldsymbol{\beta}_{\boldsymbol{TM}}$**^2^** | $\boldsymbol{\alpha}_{\boldsymbol{M}\boldsymbol{X}_{\boldsymbol{1}}}$**^3^** | $\boldsymbol{\beta}_{\boldsymbol{M}\boldsymbol{X}_{\boldsymbol{1}}}$**^4^** | **M** | $\mathbf{SE}_{\mathbf{E}}$ | $\mathbf{SE}_{\mathbf{M}}$ | **M** | $\mathbf{SE}_{\mathbf{E}}$ | $\mathbf{SE}_{\mathbf{M}}$ | **M** | $\mathbf{SE}_{\mathbf{E}}$ | $\mathbf{SE}_{\mathbf{M}}$ | **p^5^** |
| **0.3** | **0** | **0** | 1.50 | 0.02 | 0.04 | 1.81 | 0.12 | 0.13 | 0.31 | 0.13 | 0.13 | 0.016 |
| **0.3** | **0** | **0.2** | 1.49 | 0.02 | 0.04 | 1.88 | 0.13 | 0.13 | 0.38 | 0.14 | 0.13 | <0.001 |
| **0.3** | **0** | **0.4** | 1.49 | 0.02 | 0.04 | 1.93 | 0.15 | 0.13 | 0.44 | 0.16 | 0.14 | <0.001 |
| **0.3** | **0.1** | **0** | 1.50 | 0.02 | 0.04 | 1.82 | 0.13 | 0.13 | 0.33 | 0.14 | 0.13 | <0.001 |
| **0.3** | **0.1** | **0.2** | 1.49 | 0.02 | 0.04 | 1.88 | 0.13 | 0.13 | 0.39 | 0.14 | 0.13 | <0.001 |
| **0.3** | **0.1** | **0.4** | 1.49 | 0.02 | 0.04 | 1.96 | 0.15 | 0.13 | 0.47 | 0.16 | 0.13 | <0.001 |
| **0.3** | **0.2** | **0** | 1.50 | 0.02 | 0.04 | 1.81 | 0.13 | 0.13 | 0.31 | 0.14 | 0.13 | 0.111 |
| **0.3** | **0.2** | **0.2** | 1.49 | 0.02 | 0.04 | 1.90 | 0.12 | 0.13 | 0.4 | 0.14 | 0.13 | <0.001 |
| **0.3** | **0.2** | **0.4** | 1.49 | 0.02 | 0.04 | 1.99 | 0.14 | 0.13 | 0.51 | 0.15 | 0.14 | <0.001 |
| **0.6** | **0** | **0** | 1.50 | 0.02 | 0.04 | 2.12 | 0.13 | 0.13 | 0.62 | 0.14 | 0.13 | 0.002 |
| **0.6** | **0** | **0.2** | 1.49 | 0.02 | 0.04 | 2.18 | 0.13 | 0.13 | 0.68 | 0.14 | 0.13 | <0.001 |
| **0.6** | **0** | **0.4** | 1.49 | 0.02 | 0.04 | 2.22 | 0.15 | 0.13 | 0.73 | 0.16 | 0.14 | <0.001 |
| **0.6** | **0.1** | **0** | 1.50 | 0.02 | 0.04 | 2.12 | 0.13 | 0.13 | 0.62 | 0.14 | 0.13 | 0.010 |
| **0.6** | **0.1** | **0.2** | 1.49 | 0.02 | 0.04 | 2.19 | 0.13 | 0.13 | 0.7 | 0.15 | 0.13 | <0.001 |
| **0.6** | **0.1** | **0.4** | 1.49 | 0.02 | 0.04 | 2.26 | 0.14 | 0.13 | 0.77 | 0.15 | 0.13 | <0.001 |
| **0.6** | **0.2** | **0** | 1.50 | 0.02 | 0.04 | 2.12 | 0.13 | 0.13 | 0.62 | 0.14 | 0.13 | 0.002 |
| **0.6** | **0.2** | **0.2** | 1.49 | 0.02 | 0.04 | 2.2 | 0.13 | 0.13 | 0.71 | 0.15 | 0.13 | <0.001 |
| **0.6** | **0.2** | **0.4** | 1.49 | 0.02 | 0.04 | 2.29 | 0.14 | 0.13 | 0.8 | 0.15 | 0.14 | <0.001 |
|  |  |  | **Confounding model b** | | | | | | | | | |
|  |  |  | ${\hat{\boldsymbol{\beta}}}_{\boldsymbol{T\vert M=0}}$ | | | ${\hat{\boldsymbol{\beta}}}_{\boldsymbol{T\vert M=1}}$ | | | ${\hat{\boldsymbol{\beta}}}_{\boldsymbol{TM}}$ | | | |
| $\boldsymbol{\beta}_{\boldsymbol{TM}}$**^2^** | $\boldsymbol{\alpha}_{\boldsymbol{M}\boldsymbol{X}_{\boldsymbol{1}}}$**^3^** | $\boldsymbol{\beta}_{\boldsymbol{M}\boldsymbol{X}_{\boldsymbol{1}}}$**^4^** | **M** | $\mathbf{SE}_{\mathbf{E}}$ | $\mathbf{SE}_{\mathbf{M}}$ | **M** | $\mathbf{SE}_{\mathbf{E}}$ | $\mathbf{SE}_{\mathbf{M}}$ | **M** | $\mathbf{SE}_{\mathbf{E}}$ | $\mathbf{SE}_{\mathbf{M}}$ | **p^5^** |
| **0.3** | **0** | **0** | 1.51 | 0.02 | 0.04 | 1.76 | 0.17 | 0.13 | 0.25 | 0.18 | 0.13 | <0.001 |
| **0.3** | **0** | **0.2** | 1.50 | 0.02 | 0.04 | 1.81 | 0.15 | 0.13 | 0.31 | 0.17 | 0.14 | 0.084 |
| **0.3** | **0** | **0.4** | 1.49 | 0.02 | 0.04 | 1.87 | 0.15 | 0.13 | 0.37 | 0.17 | 0.14 | <0.001 |
| **0.3** | **0.1** | **0** | 1.51 | 0.02 | 0.04 | 1.72 | 0.19 | 0.13 | 0.21 | 0.21 | 0.14 | <0.001 |
| **0.3** | **0.1** | **0.2** | 1.50 | 0.02 | 0.04 | 1.78 | 0.19 | 0.13 | 0.28 | 0.2 | 0.14 | 0.011 |
| **0.3** | **0.1** | **0.4** | 1.49 | 0.02 | 0.04 | 1.85 | 0.17 | 0.13 | 0.36 | 0.19 | 0.13 | <0.001 |
| **0.3** | **0.2** | **0** | 1.52 | 0.03 | 0.04 | 1.64 | 0.24 | 0.13 | 0.12 | 0.26 | 0.14 | <0.001 |
| **0.3** | **0.2** | **0.2** | 1.51 | 0.02 | 0.04 | 1.72 | 0.20 | 0.13 | 0.22 | 0.22 | 0.13 | <0.001 |
| **0.3** | **0.2** | **0.4** | 1.50 | 0.02 | 0.04 | 1.81 | 0.20 | 0.13 | 0.31 | 0.22 | 0.14 | 0.610 |
| **0.6** | **0** | **0** | 1.51 | 0.02 | 0.04 | 2.06 | 0.18 | 0.13 | 0.55 | 0.19 | 0.13 | <0.001 |
| **0.6** | **0** | **0.2** | 1.50 | 0.02 | 0.04 | 2.11 | 0.16 | 0.13 | 0.61 | 0.17 | 0.14 | 0.124 |
| **0.6** | **0** | **0.4** | 1.49 | 0.02 | 0.04 | 2.15 | 0.17 | 0.13 | 0.66 | 0.18 | 0.14 | <0.001 |
| **0.6** | **0.1** | **0** | 1.51 | 0.02 | 0.04 | 2.02 | 0.20 | 0.13 | 0.51 | 0.22 | 0.14 | <0.001 |
| **0.6** | **0.1** | **0.2** | 1.50 | 0.02 | 0.04 | 2.08 | 0.19 | 0.13 | 0.58 | 0.21 | 0.13 | 0.072 |
| **0.6** | **0.1** | **0.4** | 1.50 | 0.02 | 0.04 | 2.14 | 0.17 | 0.13 | 0.64 | 0.19 | 0.13 | <0.001 |
| **0.6** | **0.2** | **0** | 1.51 | 0.03 | 0.04 | 1.96 | 0.23 | 0.13 | 0.44 | 0.25 | 0.14 | <0.001 |
| **0.6** | **0.2** | **0.2** | 1.51 | 0.03 | 0.04 | 2.03 | 0.22 | 0.13 | 0.52 | 0.24 | 0.14 | <0.001 |
| **0.6** | **0.2** | **0.4** | 1.50 | 0.02 | 0.04 | 2.10 | 0.20 | 0.13 | 0.60 | 0.22 | 0.14 | 0.836 |
|  |  |  | **Confounding model c** | | | | | | | | | |
|  |  |  | ${\hat{\boldsymbol{\beta}}}_{\boldsymbol{T\vert M=0}}$ | | | ${\hat{\boldsymbol{\beta}}}_{\boldsymbol{T\vert M=1}}$ | | | ${\hat{\boldsymbol{\beta}}}_{\boldsymbol{TM}}$ | | | |
| $\boldsymbol{\beta}_{\boldsymbol{TM}}$**^2^** | $\boldsymbol{\alpha}_{\boldsymbol{M}\boldsymbol{X}_{\boldsymbol{1}}}$**^3^** | $\boldsymbol{\beta}_{\boldsymbol{M}\boldsymbol{X}_{\boldsymbol{1}}}$**^4^** | **M** | $\mathbf{SE}_{\mathbf{E}}$ | $\mathbf{SE}_{\mathbf{M}}$ | **M** | $\mathbf{SE}_{\mathbf{E}}$ | $\mathbf{SE}_{\mathbf{M}}$ | **M** | $\mathbf{SE}_{\mathbf{E}}$ | $\mathbf{SE}_{\mathbf{M}}$ | **p^5^** |
| **0.3** | **0** | **0** | 1.53 | 0.03 | 0.05 | 1.49 | 0.25 | 0.14 | -0.04 | 0.27 | 0.14 | <0.001 |
| **0.3** | **0** | **0.2** | 1.53 | 0.02 | 0.05 | 1.54 | 0.23 | 0.14 | 0.01 | 0.25 | 0.15 | <0.001 |
| **0.3** | **0** | **0.4** | 1.52 | 0.03 | 0.05 | 1.59 | 0.24 | 0.14 | 0.07 | 0.26 | 0.15 | <0.001 |
| **0.3** | **0.1** | **0** | 1.53 | 0.03 | 0.05 | 1.45 | 0.26 | 0.14 | -0.08 | 0.27 | 0.15 | <0.001 |
| **0.3** | **0.1** | **0.2** | 1.53 | 0.03 | 0.05 | 1.52 | 0.25 | 0.14 | -0.01 | 0.27 | 0.15 | <0.001 |
| **0.3** | **0.1** | **0.4** | 1.52 | 0.03 | 0.05 | 1.57 | 0.25 | 0.14 | 0.05 | 0.28 | 0.15 | <0.001 |
| **0.3** | **0.2** | **0** | 1.54 | 0.03 | 0.05 | 1.38 | 0.28 | 0.14 | -0.16 | 0.30 | 0.15 | <0.001 |
| **0.3** | **0.2** | **0.2** | 1.53 | 0.03 | 0.05 | 1.46 | 0.26 | 0.14 | -0.07 | 0.28 | 0.14 | <0.001 |
| **0.3** | **0.2** | **0.4** | 1.52 | 0.03 | 0.05 | 1.55 | 0.26 | 0.14 | 0.02 | 0.28 | 0.15 | <0.001 |
| **0.6** | **0** | **0** | 1.53 | 0.03 | 0.04 | 1.79 | 0.25 | 0.14 | 0.26 | 0.27 | 0.14 | <0.001 |
| **0.6** | **0** | **0.2** | 1.53 | 0.03 | 0.05 | 1.81 | 0.25 | 0.14 | 0.28 | 0.27 | 0.15 | <0.001 |
| **0.6** | **0** | **0.4** | 1.52 | 0.03 | 0.05 | 1.88 | 0.25 | 0.14 | 0.36 | 0.27 | 0.15 | <0.001 |
| **0.6** | **0.1** | **0** | 1.53 | 0.03 | 0.05 | 1.76 | 0.26 | 0.14 | 0.23 | 0.29 | 0.15 | <0.001 |
| **0.6** | **0.1** | **0.2** | 1.53 | 0.03 | 0.05 | 1.81 | 0.26 | 0.14 | 0.29 | 0.29 | 0.15 | <0.001 |
| **0.6** | **0.1** | **0.4** | 1.52 | 0.03 | 0.05 | 1.88 | 0.25 | 0.14 | 0.35 | 0.27 | 0.14 | <0.001 |
| **0.6** | **0.2** | **0** | 1.54 | 0.03 | 0.05 | 1.71 | 0.28 | 0.14 | 0.17 | 0.31 | 0.15 | <0.001 |
| **0.6** | **0.2** | **0.2** | 1.53 | 0.03 | 0.05 | 1.77 | 0.27 | 0.14 | 0.24 | 0.29 | 0.15 | <0.001 |
| **0.6** | **0.2** | **0.4** | 1.52 | 0.03 | 0.05 | 1.86 | 0.26 | 0.14 | 0.34 | 0.28 | 0.15 | <0.001 |
|  |  |  | **Confounding model d** | | | | | | | | | |
|  |  |  | ${\hat{\boldsymbol{\beta}}}_{\boldsymbol{T\vert M=0}}$ | | | ${\hat{\boldsymbol{\beta}}}_{\boldsymbol{T\vert M=1}}$ | | | ${\hat{\boldsymbol{\beta}}}_{\boldsymbol{TM}}$ | | | |
| $\boldsymbol{\beta}_{\boldsymbol{TM}}$**^2^** | $\boldsymbol{\alpha}_{\boldsymbol{M}\boldsymbol{X}_{\boldsymbol{1}}}$**^3^** | $\boldsymbol{\beta}_{\boldsymbol{M}\boldsymbol{X}_{\boldsymbol{1}}}$**^4^** | **M** | $\mathbf{SE}_{\mathbf{E}}$ | $\mathbf{SE}_{\mathbf{M}}$ | **M** | $\mathbf{SE}_{\mathbf{E}}$ | $\mathbf{SE}_{\mathbf{M}}$ | **M** | $\mathbf{SE}_{\mathbf{E}}$ | $\mathbf{SE}_{\mathbf{M}}$ | **p^5^** |
| **0.3** | **0** | **0** | 1.50 | 0.01 | 0.04 | 1.80 | 0.05 | 0.18 | 0.30 | 0.05 | 0.19 | 0.180 |
| **0.3** | **0** | **0.2** | 1.50 | 0.01 | 0.04 | 1.80 | 0.05 | 0.20 | 0.30 | 0.05 | 0.20 | 0.372 |
| **0.3** | **0** | **0.4** | 1.50 | 0.01 | 0.04 | 1.80 | 0.05 | 0.22 | 0.30 | 0.05 | 0.22 | 0.661 |
| **0.3** | **0.1** | **0** | 1.50 | 0.01 | 0.04 | 1.80 | 0.05 | 0.18 | 0.30 | 0.05 | 0.18 | 0.342 |
| **0.3** | **0.1** | **0.2** | 1.50 | 0.01 | 0.04 | 1.80 | 0.05 | 0.19 | 0.30 | 0.05 | 0.20 | 0.648 |
| **0.3** | **0.1** | **0.4** | 1.50 | 0.01 | 0.04 | 1.80 | 0.05 | 0.21 | 0.30 | 0.05 | 0.21 | 0.999 |
| **0.3** | **0.2** | **0** | 1.50 | 0.01 | 0.04 | 1.80 | 0.05 | 0.18 | 0.30 | 0.05 | 0.18 | 0.999 |
| **0.3** | **0.2** | **0.2** | 1.50 | 0.02 | 0.04 | 1.81 | 0.05 | 0.19 | 0.31 | 0.05 | 0.20 | 0.001 |
| **0.3** | **0.2** | **0.4** | 1.50 | 0.01 | 0.04 | 1.80 | 0.05 | 0.20 | 0.30 | 0.05 | 0.21 | 0.999 |
| **0.6** | **0** | **0** | 1.50 | 0.02 | 0.04 | 2.10 | 0.05 | 0.18 | 0.60 | 0.05 | 0.19 | 0.999 |
| **0.6** | **0** | **0.2** | 1.50 | 0.01 | 0.04 | 2.10 | 0.05 | 0.20 | 0.60 | 0.05 | 0.20 | 0.999 |
| **0.6** | **0** | **0.4** | 1.50 | 0.01 | 0.04 | 2.10 | 0.05 | 0.22 | 0.60 | 0.05 | 0.23 | 0.648 |
| **0.6** | **0.1** | **0** | 1.50 | 0.01 | 0.04 | 2.10 | 0.05 | 0.18 | 0.60 | 0.05 | 0.19 | 0.999 |
| **0.6** | **0.1** | **0.2** | 1.50 | 0.01 | 0.04 | 2.10 | 0.05 | 0.19 | 0.60 | 0.05 | 0.20 | 0.655 |
| **0.6** | **0.1** | **0.4** | 1.50 | 0.01 | 0.04 | 2.10 | 0.05 | 0.21 | 0.60 | 0.05 | 0.22 | 0.198 |
| **0.6** | **0.2** | **0** | 1.50 | 0.01 | 0.04 | 2.10 | 0.05 | 0.18 | 0.60 | 0.05 | 0.18 | 0.198 |
| **0.6** | **0.2** | **0.2** | 1.50 | 0.01 | 0.04 | 2.10 | 0.05 | 0.19 | 0.60 | 0.05 | 0.19 | 0.655 |
| **0.6** | **0.2** | **0.4** | 1.50 | 0.01 | 0.04 | 2.10 | 0.05 | 0.20 | 0.60 | 0.05 | 0.21 | 0.999 |

^1^Confounding models: (A) adjusting for no moderator-confounders interactions, (B) adjusting for the one moderator-confounder interaction, (C) adjusting for all possible moderator-confounder interactions, (D) subgroup-specific confounding adjustment.
^2^$\beta_{TM}$: true magnitude of treatment effect modification
^3^$\alpha_{MX_{1}}$: magnitude of moderator-confounder interaction on treatment receipt
^4^$\beta_{MX_{1}}$: magnitude of moderator-confounder interaction on outcome
^5^p-value from a one-sample t-test comparing $\hat{\beta}_{TM}$ with the true value (0.3 or 0.6)

Table S7: Mean (M) and standard deviation (SD) of subgroup-specific treatment effects ($\hat{\beta}_{T|M=1}$ and $\hat{\beta}_{T|M=0}$) and treatment effect modification ($\hat{\beta}_{TM}$) estimated via inverse probability of treatment weighting from the 500 simulated datasets where the moderator had a prevalence of 0.1

|  |  |  | **Confounding model a** | | | | | | | | | |
| --- | --- | --- | --- | --- | --- | --- | --- | --- | --- | --- | --- | --- |
|  |  |  | ${\hat{\boldsymbol{\beta}}}_{\boldsymbol{T\vert M=0}}$ | | | ${\hat{\boldsymbol{\beta}}}_{\boldsymbol{T\vert M=1}}$ | | | ${\hat{\boldsymbol{\beta}}}_{\boldsymbol{TM}}$ | | | |
| $\boldsymbol{\beta}_{\boldsymbol{TM}}$**^2^** | $\boldsymbol{\alpha}_{\boldsymbol{M}\boldsymbol{X}_{\boldsymbol{1}}}$**^3^** | $\boldsymbol{\beta}_{\boldsymbol{M}\boldsymbol{X}_{\boldsymbol{1}}}$**^4^** | **M** | $\mathbf{SE}_{\mathbf{E}}$ | $\mathbf{SE}_{\mathbf{M}}$ | **M** | $\mathbf{SE}_{\mathbf{E}}$ | $\mathbf{SE}_{\mathbf{M}}$ | **M** | $\mathbf{SE}_{\mathbf{E}}$ | $\mathbf{SE}_{\mathbf{M}}$ | **p^5^** |
| **0.3** | **0** | **0** | 1.50 | 0.03 | 0.07 | 1.79 | 0.22 | 0.23 | 0.29 | 0.25 | 0.24 | 0.414 |
| **0.3** | **0** | **0.2** | 1.50 | 0.03 | 0.07 | 1.82 | 0.25 | 0.25 | 0.32 | 0.27 | 0.26 | 0.213 |
| **0.3** | **0** | **0.4** | 1.50 | 0.03 | 0.07 | 1.82 | 0.27 | 0.28 | 0.32 | 0.29 | 0.29 | 0.117 |
| **0.3** | **0.1** | **0** | 1.49 | 0.03 | 0.07 | 1.87 | 0.22 | 0.22 | 0.37 | 0.24 | 0.24 | <0.001 |
| **0.3** | **0.1** | **0.2** | 1.50 | 0.03 | 0.07 | 1.87 | 0.25 | 0.25 | 0.37 | 0.27 | 0.26 | <0.001 |
| **0.3** | **0.1** | **0.4** | 1.49 | 0.03 | 0.07 | 1.89 | 0.27 | 0.28 | 0.40 | 0.29 | 0.29 | <0.001 |
| **0.3** | **0.2** | **0** | 1.49 | 0.03 | 0.07 | 1.91 | 0.22 | 0.22 | 0.42 | 0.24 | 0.23 | <0.001 |
| **0.3** | **0.2** | **0.2** | 1.49 | 0.03 | 0.07 | 1.93 | 0.24 | 0.25 | 0.44 | 0.27 | 0.26 | <0.001 |
| **0.3** | **0.2** | **0.4** | 1.49 | 0.03 | 0.07 | 1.99 | 0.27 | 0.28 | 0.50 | 0.29 | 0.29 | <0.001 |
| **0.6** | **0** | **0** | 1.50 | 0.03 | 0.07 | 2.11 | 0.23 | 0.23 | 0.61 | 0.25 | 0.24 | 0.370 |
| **0.6** | **0** | **0.2** | 1.50 | 0.03 | 0.07 | 2.13 | 0.25 | 0.25 | 0.64 | 0.27 | 0.26 | 0.003 |
| **0.6** | **0** | **0.4** | 1.50 | 0.03 | 0.07 | 2.10 | 0.27 | 0.28 | 0.60 | 0.29 | 0.29 | 0.875 |
| **0.6** | **0.1** | **0** | 1.50 | 0.03 | 0.07 | 2.14 | 0.22 | 0.22 | 0.65 | 0.24 | 0.24 | <0.001 |
| **0.6** | **0.1** | **0.2** | 1.50 | 0.03 | 0.07 | 2.17 | 0.24 | 0.25 | 0.67 | 0.27 | 0.26 | <0.001 |
| **0.6** | **0.1** | **0.4** | 1.50 | 0.03 | 0.07 | 2.19 | 0.26 | 0.28 | 0.70 | 0.28 | 0.29 | <0.001 |
| **0.6** | **0.2** | **0** | 1.49 | 0.03 | 0.07 | 2.20 | 0.23 | 0.22 | 0.71 | 0.25 | 0.24 | <0.001 |
| **0.6** | **0.2** | **0.2** | 1.49 | 0.03 | 0.07 | 2.23 | 0.25 | 0.25 | 0.74 | 0.27 | 0.26 | <0.001 |
| **0.6** | **0.2** | **0.4** | 1.49 | 0.03 | 0.07 | 2.26 | 0.27 | 0.27 | 0.77 | 0.29 | 0.28 | <0.001 |
|  |  |  | **Confounding model b** | | | | | | | | | |
|  |  |  | ${\hat{\boldsymbol{\beta}}}_{\boldsymbol{T\vert M=0}}$ | | | ${\hat{\boldsymbol{\beta}}}_{\boldsymbol{T\vert M=1}}$ | | | ${\hat{\boldsymbol{\beta}}}_{\boldsymbol{TM}}$ | | | |
| $\boldsymbol{\beta}_{\boldsymbol{TM}}$**^2^** | $\boldsymbol{\alpha}_{\boldsymbol{M}\boldsymbol{X}_{\boldsymbol{1}}}$**^3^** | $\boldsymbol{\beta}_{\boldsymbol{M}\boldsymbol{X}_{\boldsymbol{1}}}$**^4^** | **M** | $\mathbf{SE}_{\mathbf{E}}$ | $\mathbf{SE}_{\mathbf{M}}$ | **M** | $\mathbf{SE}_{\mathbf{E}}$ | $\mathbf{SE}_{\mathbf{M}}$ | **M** | $\mathbf{SE}_{\mathbf{E}}$ | $\mathbf{SE}_{\mathbf{M}}$ | **p^5^** |
| **0.3** | **0** | **0** | 1.50 | 0.03 | 0.07 | 1.79 | 0.19 | 0.23 | 0.29 | 0.21 | 0.24 | 0.343 |
| **0.3** | **0** | **0.2** | 1.50 | 0.03 | 0.07 | 1.82 | 0.20 | 0.25 | 0.32 | 0.22 | 0.27 | 0.083 |
| **0.3** | **0** | **0.4** | 1.50 | 0.03 | 0.07 | 1.81 | 0.20 | 0.29 | 0.31 | 0.21 | 0.30 | 0.347 |
| **0.3** | **0.1** | **0** | 1.50 | 0.03 | 0.07 | 1.83 | 0.20 | 0.23 | 0.33 | 0.21 | 0.24 | 0.003 |
| **0.3** | **0.1** | **0.2** | 1.50 | 0.03 | 0.07 | 1.81 | 0.20 | 0.26 | 0.31 | 0.22 | 0.27 | 0.264 |
| **0.3** | **0.1** | **0.4** | 1.50 | 0.03 | 0.07 | 1.82 | 0.22 | 0.29 | 0.32 | 0.24 | 0.30 | 0.030 |
| **0.3** | **0.2** | **0** | 1.50 | 0.03 | 0.07 | 1.83 | 0.21 | 0.24 | 0.33 | 0.23 | 0.25 | 0.012 |
| **0.3** | **0.2** | **0.2** | 1.50 | 0.03 | 0.07 | 1.81 | 0.21 | 0.27 | 0.31 | 0.23 | 0.28 | 0.165 |
| **0.3** | **0.2** | **0.4** | 1.50 | 0.03 | 0.07 | 1.82 | 0.22 | 0.31 | 0.33 | 0.24 | 0.32 | 0.019 |
| **0.6** | **0** | **0** | 1.50 | 0.03 | 0.07 | 2.11 | 0.21 | 0.23 | 0.61 | 0.23 | 0.24 | 0.238 |
| **0.6** | **0** | **0.2** | 1.50 | 0.03 | 0.07 | 2.13 | 0.21 | 0.26 | 0.63 | 0.23 | 0.27 | 0.004 |
| **0.6** | **0** | **0.4** | 1.50 | 0.03 | 0.07 | 2.10 | 0.21 | 0.29 | 0.60 | 0.22 | 0.30 | 0.689 |
| **0.6** | **0.1** | **0** | 1.50 | 0.03 | 0.07 | 2.11 | 0.20 | 0.23 | 0.61 | 0.22 | 0.24 | 0.546 |
| **0.6** | **0.1** | **0.2** | 1.50 | 0.03 | 0.07 | 2.11 | 0.21 | 0.26 | 0.61 | 0.23 | 0.27 | 0.500 |
| **0.6** | **0.1** | **0.4** | 1.50 | 0.03 | 0.07 | 2.12 | 0.20 | 0.29 | 0.62 | 0.21 | 0.30 | 0.092 |
| **0.6** | **0.2** | **0** | 1.50 | 0.03 | 0.07 | 2.12 | 0.21 | 0.24 | 0.62 | 0.23 | 0.25 | 0.122 |
| **0.6** | **0.2** | **0.2** | 1.50 | 0.03 | 0.07 | 2.11 | 0.22 | 0.26 | 0.61 | 0.23 | 0.27 | 0.213 |
| **0.6** | **0.2** | **0.4** | 1.50 | 0.02 | 0.07 | 2.12 | 0.21 | 0.29 | 0.62 | 0.22 | 0.30 | 0.131 |
|  |  |  | **Confounding model c** | | | | | | | | | |
|  |  |  | ${\hat{\boldsymbol{\beta}}}_{\boldsymbol{T\vert M=0}}$ | | | ${\hat{\boldsymbol{\beta}}}_{\boldsymbol{T\vert M=1}}$ | | | ${\hat{\boldsymbol{\beta}}}_{\boldsymbol{TM}}$ | | | |
| $\boldsymbol{\beta}_{\boldsymbol{TM}}$**^2^** | $\boldsymbol{\alpha}_{\boldsymbol{M}\boldsymbol{X}_{\boldsymbol{1}}}$**^3^** | $\boldsymbol{\beta}_{\boldsymbol{M}\boldsymbol{X}_{\boldsymbol{1}}}$**^4^** | **M** | $\mathbf{SE}_{\mathbf{E}}$ | $\mathbf{SE}_{\mathbf{M}}$ | **M** | $\mathbf{SE}_{\mathbf{E}}$ | $\mathbf{SE}_{\mathbf{M}}$ | **M** | $\mathbf{SE}_{\mathbf{E}}$ | $\mathbf{SE}_{\mathbf{M}}$ | **p^5^** |
| **0.3** | **0** | **0** | 1.50 | 0.02 | 0.07 | 1.81 | 0.09 | 0.24 | 0.31 | 0.09 | 0.25 | 0.027 |
| **0.3** | **0** | **0.2** | 1.50 | 0.02 | 0.07 | 1.82 | 0.10 | 0.26 | 0.32 | 0.10 | 0.27 | <0.001 |
| **0.3** | **0** | **0.4** | 1.50 | 0.02 | 0.07 | 1.80 | 0.12 | 0.30 | 0.30 | 0.12 | 0.31 | 0.847 |
| **0.3** | **0.1** | **0** | 1.50 | 0.02 | 0.07 | 1.80 | 0.12 | 0.25 | 0.30 | 0.12 | 0.26 | 0.567 |
| **0.3** | **0.1** | **0.2** | 1.50 | 0.02 | 0.07 | 1.82 | 0.10 | 0.27 | 0.32 | 0.10 | 0.28 | 0.001 |
| **0.3** | **0.1** | **0.4** | 1.50 | 0.02 | 0.07 | 1.81 | 0.11 | 0.30 | 0.31 | 0.11 | 0.31 | 0.070 |
| **0.3** | **0.2** | **0** | 1.50 | 0.02 | 0.07 | 1.82 | 0.11 | 0.25 | 0.32 | 0.11 | 0.26 | 0.003 |
| **0.3** | **0.2** | **0.2** | 1.50 | 0.02 | 0.07 | 1.81 | 0.13 | 0.28 | 0.32 | 0.13 | 0.29 | 0.011 |
| **0.3** | **0.2** | **0.4** | 1.50 | 0.02 | 0.07 | 1.82 | 0.15 | 0.32 | 0.32 | 0.15 | 0.33 | 0.025 |
| **0.6** | **0** | **0** | 1.50 | 0.02 | 0.07 | 2.11 | 0.09 | 0.24 | 0.61 | 0.09 | 0.25 | 0.093 |
| **0.6** | **0** | **0.2** | 1.50 | 0.02 | 0.07 | 2.11 | 0.11 | 0.27 | 0.61 | 0.11 | 0.28 | 0.039 |
| **0.6** | **0** | **0.4** | 1.50 | 0.02 | 0.07 | 2.11 | 0.10 | 0.30 | 0.61 | 0.10 | 0.31 | 0.005 |
| **0.6** | **0.1** | **0** | 1.50 | 0.02 | 0.07 | 2.11 | 0.10 | 0.24 | 0.61 | 0.10 | 0.25 | 0.007 |
| **0.6** | **0.1** | **0.2** | 1.50 | 0.02 | 0.07 | 2.11 | 0.11 | 0.27 | 0.61 | 0.11 | 0.28 | 0.023 |
| **0.6** | **0.1** | **0.4** | 1.50 | 0.02 | 0.07 | 2.12 | 0.11 | 0.30 | 0.62 | 0.11 | 0.31 | <0.001 |
| **0.6** | **0.2** | **0** | 1.50 | 0.02 | 0.07 | 2.11 | 0.11 | 0.25 | 0.61 | 0.11 | 0.26 | 0.044 |
| **0.6** | **0.2** | **0.2** | 1.50 | 0.02 | 0.07 | 2.12 | 0.11 | 0.27 | 0.62 | 0.11 | 0.28 | 0.001 |
| **0.6** | **0.2** | **0.4** | 1.50 | 0.02 | 0.07 | 2.11 | 0.14 | 0.31 | 0.61 | 0.14 | 0.32 | 0.025 |
|  |  |  | **Confounding model d** | | | | | | | | | |
|  |  |  | ${\hat{\boldsymbol{\beta}}}_{\boldsymbol{T\vert M=0}}$ | | | ${\hat{\boldsymbol{\beta}}}_{\boldsymbol{T\vert M=1}}$ | | | ${\hat{\boldsymbol{\beta}}}_{\boldsymbol{TM}}$ | | | |
| $\boldsymbol{\beta}_{\boldsymbol{TM}}$**^2^** | $\boldsymbol{\alpha}_{\boldsymbol{M}\boldsymbol{X}_{\boldsymbol{1}}}$**^3^** | $\boldsymbol{\beta}_{\boldsymbol{M}\boldsymbol{X}_{\boldsymbol{1}}}$**^4^** | **M** | $\mathbf{SE}_{\mathbf{E}}$ | $\mathbf{SE}_{\mathbf{M}}$ | **M** | $\mathbf{SE}_{\mathbf{E}}$ | $\mathbf{SE}_{\mathbf{M}}$ | **M** | $\mathbf{SE}_{\mathbf{E}}$ | $\mathbf{SE}_{\mathbf{M}}$ | **p^5^** |
| **0.3** | **0** | **0** | 1.50 | 0.02 | 0.07 | 1.81 | 0.09 | 0.24 | 0.31 | 0.09 | 0.25 | 0.027 |
| **0.3** | **0** | **0.2** | 1.50 | 0.02 | 0.07 | 1.82 | 0.10 | 0.27 | 0.32 | 0.10 | 0.28 | <0.001 |
| **0.3** | **0** | **0.4** | 1.50 | 0.02 | 0.07 | 1.80 | 0.12 | 0.31 | 0.30 | 0.12 | 0.32 | 0.847 |
| **0.3** | **0.1** | **0** | 1.50 | 0.02 | 0.07 | 1.80 | 0.12 | 0.25 | 0.30 | 0.12 | 0.26 | 0.567 |
| **0.3** | **0.1** | **0.2** | 1.50 | 0.02 | 0.07 | 1.82 | 0.10 | 0.27 | 0.32 | 0.10 | 0.28 | 0.001 |
| **0.3** | **0.1** | **0.4** | 1.50 | 0.02 | 0.07 | 1.81 | 0.11 | 0.31 | 0.31 | 0.11 | 0.32 | 0.070 |
| **0.3** | **0.2** | **0** | 1.50 | 0.02 | 0.07 | 1.82 | 0.11 | 0.25 | 0.32 | 0.11 | 0.26 | 0.003 |
| **0.3** | **0.2** | **0.2** | 1.50 | 0.02 | 0.07 | 1.81 | 0.13 | 0.28 | 0.32 | 0.13 | 0.29 | 0.011 |
| **0.3** | **0.2** | **0.4** | 1.50 | 0.02 | 0.07 | 1.82 | 0.15 | 0.32 | 0.32 | 0.15 | 0.33 | 0.025 |
| **0.6** | **0** | **0** | 1.50 | 0.02 | 0.07 | 2.11 | 0.09 | 0.24 | 0.61 | 0.09 | 0.25 | 0.093 |
| **0.6** | **0** | **0.2** | 1.50 | 0.02 | 0.07 | 2.11 | 0.11 | 0.27 | 0.61 | 0.11 | 0.28 | 0.039 |
| **0.6** | **0** | **0.4** | 1.50 | 0.02 | 0.07 | 2.11 | 0.10 | 0.30 | 0.61 | 0.10 | 0.31 | 0.005 |
| **0.6** | **0.1** | **0** | 1.50 | 0.02 | 0.07 | 2.11 | 0.10 | 0.24 | 0.61 | 0.10 | 0.25 | 0.007 |
| **0.6** | **0.1** | **0.2** | 1.50 | 0.02 | 0.07 | 2.11 | 0.11 | 0.27 | 0.61 | 0.11 | 0.28 | 0.023 |
| **0.6** | **0.1** | **0.4** | 1.50 | 0.02 | 0.07 | 2.12 | 0.11 | 0.30 | 0.62 | 0.11 | 0.31 | <0.001 |
| **0.6** | **0.2** | **0** | 1.50 | 0.02 | 0.07 | 2.11 | 0.11 | 0.25 | 0.61 | 0.11 | 0.26 | 0.044 |
| **0.6** | **0.2** | **0.2** | 1.50 | 0.02 | 0.07 | 2.12 | 0.11 | 0.28 | 0.62 | 0.11 | 0.28 | 0.001 |
| **0.6** | **0.2** | **0.4** | 1.50 | 0.02 | 0.07 | 2.11 | 0.14 | 0.31 | 0.61 | 0.14 | 0.32 | 0.025 |

^1^Confounding models: (A) adjusting for no moderator-confounders interactions, (B) adjusting for the one moderator-confounder interaction, (C) adjusting for all possible moderator-confounder interactions, (D) subgroup-specific confounding adjustment.
^2^$\beta_{TM}$: true magnitude of treatment effect modification
^3^$\alpha_{MX_{1}}$: magnitude of moderator-confounder interaction on treatment receipt
^4^$\beta_{MX_{1}}$: magnitude of moderator-confounder interaction on outcome
^5^p-value from a one-sample t-test comparing $\hat{\beta}_{TM}$ with the true value (0.3 or 0.6)

Table S8: Mean (M) and standard deviation (SD) of subgroup-specific treatment effects ($\hat{\beta}_{T|M=1}$ and $\hat{\beta}_{T|M=0}$) and treatment effect modification ($\hat{\beta}_{TM}$) estimated via propensity score matching from the 500 simulated datasets where the moderator had a prevalence of 0.1

|  |  |  | **Confounding model a** | | | | | | | | | |
| --- | --- | --- | --- | --- | --- | --- | --- | --- | --- | --- | --- | --- |
|  |  |  | ${\hat{\boldsymbol{\beta}}}_{\boldsymbol{T\vert M=0}}$ | | | ${\hat{\boldsymbol{\beta}}}_{\boldsymbol{T\vert M=1}}$ | | | ${\hat{\boldsymbol{\beta}}}_{\boldsymbol{TM}}$ | | | |
| $\boldsymbol{\beta}_{\boldsymbol{TM}}$**^2^** | $\boldsymbol{\alpha}_{\boldsymbol{M}\boldsymbol{X}_{\boldsymbol{1}}}$**^3^** | $\boldsymbol{\beta}_{\boldsymbol{M}\boldsymbol{X}_{\boldsymbol{1}}}$**^4^** | **M** | $\mathbf{SE}_{\mathbf{E}}$ | $\mathbf{SE}_{\mathbf{M}}$ | **M** | $\mathbf{SE}_{\mathbf{E}}$ | $\mathbf{SE}_{\mathbf{M}}$ | **M** | $\mathbf{SE}_{\mathbf{E}}$ | $\mathbf{SE}_{\mathbf{M}}$ | **p^5^** |
| **0.3** | **0** | **0** | 1.50 | 0.05 | 0.10 | 1.82 | 0.27 | 0.27 | 0.32 | 0.30 | 0.29 | 0.104 |
| **0.3** | **0** | **0.2** | 1.50 | 0.05 | 0.10 | 1.86 | 0.28 | 0.29 | 0.36 | 0.31 | 0.31 | <0.001 |
| **0.3** | **0** | **0.4** | 1.51 | 0.05 | 0.10 | 1.84 | 0.32 | 0.34 | 0.33 | 0.35 | 0.35 | 0.039 |
| **0.3** | **0.1** | **0** | 1.49 | 0.05 | 0.10 | 1.88 | 0.26 | 0.27 | 0.39 | 0.29 | 0.29 | <0.001 |
| **0.3** | **0.1** | **0.2** | 1.49 | 0.05 | 0.10 | 1.88 | 0.30 | 0.30 | 0.39 | 0.33 | 0.32 | <0.001 |
| **0.3** | **0.1** | **0.4** | 1.49 | 0.05 | 0.10 | 1.90 | 0.33 | 0.34 | 0.41 | 0.36 | 0.35 | <0.001 |
| **0.3** | **0.2** | **0** | 1.49 | 0.05 | 0.10 | 1.92 | 0.28 | 0.27 | 0.43 | 0.32 | 0.29 | <0.001 |
| **0.3** | **0.2** | **0.2** | 1.49 | 0.05 | 0.10 | 1.94 | 0.31 | 0.31 | 0.45 | 0.34 | 0.32 | <0.001 |
| **0.3** | **0.2** | **0.4** | 1.49 | 0.05 | 0.10 | 1.99 | 0.32 | 0.34 | 0.50 | 0.35 | 0.35 | <0.001 |
| **0.6** | **0** | **0** | 1.50 | 0.05 | 0.10 | 2.12 | 0.28 | 0.28 | 0.62 | 0.31 | 0.29 | 0.124 |
| **0.6** | **0** | **0.2** | 1.50 | 0.05 | 0.10 | 2.15 | 0.30 | 0.30 | 0.66 | 0.33 | 0.32 | <0.001 |
| **0.6** | **0** | **0.4** | 1.50 | 0.05 | 0.10 | 2.14 | 0.31 | 0.34 | 0.64 | 0.34 | 0.35 | 0.021 |
| **0.6** | **0.1** | **0** | 1.50 | 0.05 | 0.10 | 2.14 | 0.26 | 0.27 | 0.64 | 0.29 | 0.29 | 0.001 |
| **0.6** | **0.1** | **0.2** | 1.50 | 0.05 | 0.10 | 2.17 | 0.29 | 0.30 | 0.67 | 0.32 | 0.32 | <0.001 |
| **0.6** | **0.1** | **0.4** | 1.50 | 0.05 | 0.10 | 2.18 | 0.32 | 0.34 | 0.69 | 0.35 | 0.36 | <0.001 |
| **0.6** | **0.2** | **0** | 1.49 | 0.05 | 0.10 | 2.20 | 0.28 | 0.27 | 0.71 | 0.31 | 0.29 | <0.001 |
| **0.6** | **0.2** | **0.2** | 1.50 | 0.06 | 0.10 | 2.21 | 0.30 | 0.31 | 0.71 | 0.33 | 0.32 | <0.001 |
| **0.6** | **0.2** | **0.4** | 1.49 | 0.05 | 0.10 | 2.28 | 0.33 | 0.33 | 0.79 | 0.36 | 0.35 | <0.001 |
|  |  |  | **Confounding model b** | | | | | | | | | |
|  |  |  | ${\hat{\boldsymbol{\beta}}}_{\boldsymbol{T\vert M=0}}$ | | | ${\hat{\boldsymbol{\beta}}}_{\boldsymbol{T\vert M=1}}$ | | | ${\hat{\boldsymbol{\beta}}}_{\boldsymbol{TM}}$ | | | |
| $\boldsymbol{\beta}_{\boldsymbol{TM}}$**^2^** | $\boldsymbol{\alpha}_{\boldsymbol{M}\boldsymbol{X}_{\boldsymbol{1}}}$**^3^** | $\boldsymbol{\beta}_{\boldsymbol{M}\boldsymbol{X}_{\boldsymbol{1}}}$**^4^** | **M** | $\mathbf{SE}_{\mathbf{E}}$ | $\mathbf{SE}_{\mathbf{M}}$ | **M** | $\mathbf{SE}_{\mathbf{E}}$ | $\mathbf{SE}_{\mathbf{M}}$ | **M** | $\mathbf{SE}_{\mathbf{E}}$ | $\mathbf{SE}_{\mathbf{M}}$ | **p^5^** |
| **0.3** | **0** | **0** | 1.50 | 0.05 | 0.10 | 1.81 | 0.25 | 0.28 | 0.31 | 0.28 | 0.29 | 0.377 |
| **0.3** | **0** | **0.2** | 1.50 | 0.05 | 0.10 | 1.83 | 0.25 | 0.30 | 0.34 | 0.27 | 0.31 | 0.003 |
| **0.3** | **0** | **0.4** | 1.50 | 0.05 | 0.10 | 1.83 | 0.29 | 0.34 | 0.33 | 0.31 | 0.35 | 0.064 |
| **0.3** | **0.1** | **0** | 1.50 | 0.05 | 0.10 | 1.84 | 0.25 | 0.27 | 0.35 | 0.27 | 0.29 | <0.001 |
| **0.3** | **0.1** | **0.2** | 1.50 | 0.05 | 0.10 | 1.83 | 0.25 | 0.30 | 0.33 | 0.27 | 0.31 | 0.015 |
| **0.3** | **0.1** | **0.4** | 1.50 | 0.05 | 0.10 | 1.84 | 0.28 | 0.34 | 0.34 | 0.31 | 0.35 | 0.007 |
| **0.3** | **0.2** | **0** | 1.50 | 0.05 | 0.10 | 1.85 | 0.26 | 0.27 | 0.35 | 0.28 | 0.29 | <0.001 |
| **0.3** | **0.2** | **0.2** | 1.50 | 0.05 | 0.10 | 1.85 | 0.27 | 0.30 | 0.35 | 0.29 | 0.32 | <0.001 |
| **0.3** | **0.2** | **0.4** | 1.50 | 0.05 | 0.10 | 1.86 | 0.27 | 0.34 | 0.36 | 0.30 | 0.35 | <0.001 |
| **0.6** | **0** | **0** | 1.50 | 0.05 | 0.10 | 2.13 | 0.25 | 0.28 | 0.63 | 0.28 | 0.29 | 0.026 |
| **0.6** | **0** | **0.2** | 1.50 | 0.05 | 0.10 | 2.14 | 0.28 | 0.30 | 0.64 | 0.30 | 0.32 | 0.001 |
| **0.6** | **0** | **0.4** | 1.50 | 0.05 | 0.10 | 2.14 | 0.27 | 0.34 | 0.64 | 0.29 | 0.35 | 0.003 |
| **0.6** | **0.1** | **0** | 1.50 | 0.05 | 0.10 | 2.12 | 0.26 | 0.28 | 0.62 | 0.28 | 0.29 | 0.056 |
| **0.6** | **0.1** | **0.2** | 1.50 | 0.05 | 0.10 | 2.14 | 0.26 | 0.30 | 0.63 | 0.29 | 0.32 | 0.009 |
| **0.6** | **0.1** | **0.4** | 1.50 | 0.05 | 0.10 | 2.15 | 0.27 | 0.34 | 0.65 | 0.29 | 0.35 | 0.001 |
| **0.6** | **0.2** | **0** | 1.50 | 0.05 | 0.10 | 2.13 | 0.25 | 0.28 | 0.63 | 0.27 | 0.30 | 0.009 |
| **0.6** | **0.2** | **0.2** | 1.51 | 0.05 | 0.10 | 2.15 | 0.27 | 0.30 | 0.65 | 0.30 | 0.32 | 0.001 |
| **0.6** | **0.2** | **0.4** | 1.50 | 0.05 | 0.10 | 2.14 | 0.27 | 0.34 | 0.64 | 0.29 | 0.35 | 0.002 |
|  |  |  | **Confounding model c** | | | | | | | | | |
|  |  |  | ${\hat{\boldsymbol{\beta}}}_{\boldsymbol{T\vert M=0}}$ | | | ${\hat{\boldsymbol{\beta}}}_{\boldsymbol{T\vert M=1}}$ | | | ${\hat{\boldsymbol{\beta}}}_{\boldsymbol{TM}}$ | | | |
| $\boldsymbol{\beta}_{\boldsymbol{TM}}$**^2^** | $\boldsymbol{\alpha}_{\boldsymbol{M}\boldsymbol{X}_{\boldsymbol{1}}}$**^3^** | $\boldsymbol{\beta}_{\boldsymbol{M}\boldsymbol{X}_{\boldsymbol{1}}}$**^4^** | **M** | $\mathbf{SE}_{\mathbf{E}}$ | $\mathbf{SE}_{\mathbf{M}}$ | **M** | $\mathbf{SE}_{\mathbf{E}}$ | $\mathbf{SE}_{\mathbf{M}}$ | **M** | $\mathbf{SE}_{\mathbf{E}}$ | $\mathbf{SE}_{\mathbf{M}}$ | **p^5^** |
| **0.3** | **0** | **0** | 1.50 | 0.05 | 0.10 | 1.82 | 0.21 | 0.28 | 0.32 | 0.22 | 0.30 | 0.022 |
| **0.3** | **0** | **0.2** | 1.50 | 0.05 | 0.10 | 1.86 | 0.20 | 0.31 | 0.36 | 0.21 | 0.32 | <0.001 |
| **0.3** | **0** | **0.4** | 1.50 | 0.05 | 0.10 | 1.83 | 0.24 | 0.35 | 0.33 | 0.26 | 0.37 | 0.029 |
| **0.3** | **0.1** | **0** | 1.50 | 0.05 | 0.10 | 1.84 | 0.19 | 0.28 | 0.34 | 0.20 | 0.30 | <0.001 |
| **0.3** | **0.1** | **0.2** | 1.50 | 0.05 | 0.10 | 1.84 | 0.22 | 0.32 | 0.34 | 0.23 | 0.33 | <0.001 |
| **0.3** | **0.1** | **0.4** | 1.50 | 0.05 | 0.10 | 1.86 | 0.24 | 0.34 | 0.36 | 0.26 | 0.35 | <0.001 |
| **0.3** | **0.2** | **0** | 1.50 | 0.05 | 0.10 | 1.84 | 0.20 | 0.28 | 0.34 | 0.21 | 0.30 | <0.001 |
| **0.3** | **0.2** | **0.2** | 1.50 | 0.05 | 0.10 | 1.85 | 0.23 | 0.31 | 0.35 | 0.24 | 0.33 | <0.001 |
| **0.3** | **0.2** | **0.4** | 1.50 | 0.05 | 0.10 | 1.86 | 0.25 | 0.35 | 0.36 | 0.26 | 0.36 | <0.001 |
| **0.6** | **0** | **0** | 1.50 | 0.05 | 0.10 | 2.14 | 0.20 | 0.28 | 0.64 | 0.22 | 0.30 | <0.001 |
| **0.6** | **0** | **0.2** | 1.50 | 0.05 | 0.10 | 2.13 | 0.22 | 0.32 | 0.63 | 0.24 | 0.33 | 0.009 |
| **0.6** | **0** | **0.4** | 1.50 | 0.05 | 0.10 | 2.15 | 0.25 | 0.35 | 0.65 | 0.27 | 0.36 | <0.001 |
| **0.6** | **0.1** | **0** | 1.50 | 0.05 | 0.10 | 2.14 | 0.20 | 0.28 | 0.64 | 0.21 | 0.30 | <0.001 |
| **0.6** | **0.1** | **0.2** | 1.50 | 0.05 | 0.10 | 2.13 | 0.23 | 0.31 | 0.63 | 0.24 | 0.33 | 0.002 |
| **0.6** | **0.1** | **0.4** | 1.50 | 0.05 | 0.10 | 2.16 | 0.25 | 0.34 | 0.66 | 0.26 | 0.36 | <0.001 |
| **0.6** | **0.2** | **0** | 1.50 | 0.05 | 0.10 | 2.15 | 0.20 | 0.28 | 0.65 | 0.21 | 0.30 | <0.001 |
| **0.6** | **0.2** | **0.2** | 1.50 | 0.05 | 0.10 | 2.17 | 0.21 | 0.31 | 0.67 | 0.22 | 0.32 | <0.001 |
| **0.6** | **0.2** | **0.4** | 1.50 | 0.05 | 0.10 | 2.15 | 0.23 | 0.35 | 0.65 | 0.25 | 0.36 | <0.001 |
|  |  |  | **Confounding model d** | | | | | | | | | |
|  |  |  | ${\hat{\boldsymbol{\beta}}}_{\boldsymbol{T\vert M=0}}$ | | | ${\hat{\boldsymbol{\beta}}}_{\boldsymbol{T\vert M=1}}$ | | | ${\hat{\boldsymbol{\beta}}}_{\boldsymbol{TM}}$ | | | |
| $\boldsymbol{\beta}_{\boldsymbol{TM}}$**^2^** | $\boldsymbol{\alpha}_{\boldsymbol{M}\boldsymbol{X}_{\boldsymbol{1}}}$**^3^** | $\boldsymbol{\beta}_{\boldsymbol{M}\boldsymbol{X}_{\boldsymbol{1}}}$**^4^** | **M** | $\mathbf{SE}_{\mathbf{E}}$ | $\mathbf{SE}_{\mathbf{M}}$ | **M** | $\mathbf{SE}_{\mathbf{E}}$ | $\mathbf{SE}_{\mathbf{M}}$ | **M** | $\mathbf{SE}_{\mathbf{E}}$ | $\mathbf{SE}_{\mathbf{M}}$ | **p^5^** |
| **0.3** | **0** | **0** | 1.50 | 0.04 | 0.10 | 1.82 | 0.18 | 0.28 | 0.32 | 0.18 | 0.30 | 0.029 |
| **0.3** | **0** | **0.2** | 1.50 | 0.04 | 0.10 | 1.83 | 0.18 | 0.31 | 0.33 | 0.19 | 0.32 | <0.001 |
| **0.3** | **0** | **0.4** | 1.51 | 0.04 | 0.10 | 1.83 | 0.20 | 0.35 | 0.33 | 0.21 | 0.36 | 0.003 |
| **0.3** | **0.1** | **0** | 1.50 | 0.04 | 0.10 | 1.83 | 0.16 | 0.28 | 0.33 | 0.17 | 0.30 | <0.001 |
| **0.3** | **0.1** | **0.2** | 1.50 | 0.04 | 0.10 | 1.84 | 0.21 | 0.32 | 0.33 | 0.21 | 0.33 | 0.001 |
| **0.3** | **0.1** | **0.4** | 1.50 | 0.04 | 0.10 | 1.86 | 0.18 | 0.34 | 0.36 | 0.19 | 0.35 | <0.001 |
| **0.3** | **0.2** | **0** | 1.51 | 0.04 | 0.10 | 1.84 | 0.18 | 0.28 | 0.34 | 0.18 | 0.30 | <0.001 |
| **0.3** | **0.2** | **0.2** | 1.50 | 0.04 | 0.10 | 1.87 | 0.20 | 0.32 | 0.37 | 0.20 | 0.33 | <0.001 |
| **0.3** | **0.2** | **0.4** | 1.51 | 0.04 | 0.10 | 1.86 | 0.22 | 0.35 | 0.35 | 0.23 | 0.37 | <0.001 |
| **0.6** | **0** | **0** | 1.51 | 0.04 | 0.10 | 2.13 | 0.18 | 0.28 | 0.63 | 0.18 | 0.30 | <0.001 |
| **0.6** | **0** | **0.2** | 1.50 | 0.04 | 0.10 | 2.14 | 0.19 | 0.32 | 0.64 | 0.20 | 0.33 | <0.001 |
| **0.6** | **0** | **0.4** | 1.50 | 0.04 | 0.10 | 2.13 | 0.21 | 0.35 | 0.63 | 0.22 | 0.36 | 0.002 |
| **0.6** | **0.1** | **0** | 1.50 | 0.04 | 0.10 | 2.13 | 0.17 | 0.29 | 0.63 | 0.18 | 0.30 | 0.001 |
| **0.6** | **0.1** | **0.2** | 1.50 | 0.04 | 0.10 | 2.14 | 0.19 | 0.31 | 0.64 | 0.19 | 0.32 | <0.001 |
| **0.6** | **0.1** | **0.4** | 1.51 | 0.04 | 0.10 | 2.17 | 0.21 | 0.35 | 0.66 | 0.21 | 0.36 | <0.001 |
| **0.6** | **0.2** | **0** | 1.50 | 0.04 | 0.10 | 2.14 | 0.18 | 0.29 | 0.63 | 0.18 | 0.31 | <0.001 |
| **0.6** | **0.2** | **0.2** | 1.50 | 0.04 | 0.10 | 2.16 | 0.19 | 0.31 | 0.66 | 0.19 | 0.33 | <0.001 |
| **0.6** | **0.2** | **0.4** | 1.50 | 0.04 | 0.10 | 2.16 | 0.20 | 0.34 | 0.65 | 0.20 | 0.36 | <0.001 |

^1^Confounding models: (A) adjusting for no moderator-confounders interactions, (B) adjusting for the one moderator-confounder interaction, (C) adjusting for all possible moderator-confounder interactions, (D) subgroup-specific confounding adjustment.
^2^$\beta_{TM}$: true magnitude of treatment effect modification
^3^$\alpha_{MX_{1}}$: magnitude of moderator-confounder interaction on treatment receipt
^4^$\beta_{MX_{1}}$: magnitude of moderator-confounder interaction on outcome
^5^p-value from a one-sample t-test comparing $\hat{\beta}_{TM}$ with the true value (0.3 or 0.6)
